# Glycoside-metabolizing oxidoreductase D3dgpA from human gut bacterium

**DOI:** 10.3389/fbioe.2024.1413854

**Published:** 2024-06-28

**Authors:** Heji Kim, Huynh Thi Ngoc Mi, Joong-Hoon Ahn, Jong Suk Lee, Bekir Engin Eser, Jongkeun Choi, Jaehong Han

**Affiliations:** ^1^ Metalloenzyme Research Group and Department of Plant Science and Technology, Chung-Ang University, Anseong, Republic of Korea; ^2^ Department of Integrative Bioscience and Biotechnology, Bio/Molecular Informatics Center, Konkuk University, Seoul, Republic of Korea; ^3^ Bio Industry Department, Gyeonggido Business and Science Accelerator (GBSA), Suwon, Gyeonngi-do, Republic of Korea; ^4^ Department of Biological and Chemical Engineering, Aarhus University, Aarhus, Denmark; ^5^ Department of Chemical Engineering, Chungwoon University, Incheon, Republic of Korea

**Keywords:** deglycosylation, D3dgpA, Gfo/Idh/MocA family, glycosides, NAD^+^, oxidoreductase

## Abstract

The Gfo/Idh/MocA family enzyme DgpA was known to catalyze the regiospecific oxidation of puerarin to 3”-oxo-puerarin in the presence of 3-oxo-glucose. Here, we discovered that D3dgpA, *dgpA* cloned from the human gut bacterium *Dorea* sp. MRG-IFC3, catalyzed the regiospecific oxidation of various *C*-/*O*-glycosides, including puerarin, in the presence of methyl β-D-3-oxo-glucopyranoside. While *C*-glycosides were converted to 3”- and 2”-oxo-products by D3dgpA, *O*-glycosides resulted in the formation of aglycones and hexose enediolone from the 3”-oxo-products. From DFT calculations, it was found that isomerization of 3”-oxo-puerarin to 2”-oxo-puerarin required a small activation energy of 9.86 kcal/mol, and the *O*-glycosidic bond cleavage of 3”-oxo-products was also thermodynamically favored with a small activation energy of 3.49 kcal/mol. In addition, the reaction mechanism of D3dgpA was discussed in comparison to those of Gfo/Idh/MocA and GMC family enzymes. The robust reactivity of D3dgpA was proposed as a new general route for derivatization of glycosides.

## 1 Introduction

*C*-Glycosides have been noticed as important bioactive compounds. Particularly, natural aryl *C*-glycosides are a group of natural products with the structural feature of an aromatic moiety and sugar(s) directly connected through a glycosidic C-C bond (Kitamura et al., 2018). Due to the various biological activities, such as antibacterial, antitumor, antidiabetic, and antioxidant activities (Xiao et al., 2016; Min et al., 2021; Tao et al., 2022; Xie et al., 2022), research on biosynthesis, chemical synthesis (Ni et al., 2022; Chong et al., 2023), and bioavailability (Peng et al., 2021) of *C*-glycosides has increased recently. One of the characteristics of *C*-glycosides is the inertness to biological metabolism. Only a few human gut bacteria have been reported to be able to degrade *C*-glycosides (Supplementary Table S1). Accordingly, the metabolism of *C*-glycosides in the human body has drawn a great deal of interest (Mori et al., 2021; Choi et al., 2023).

**FIGURE 1:**
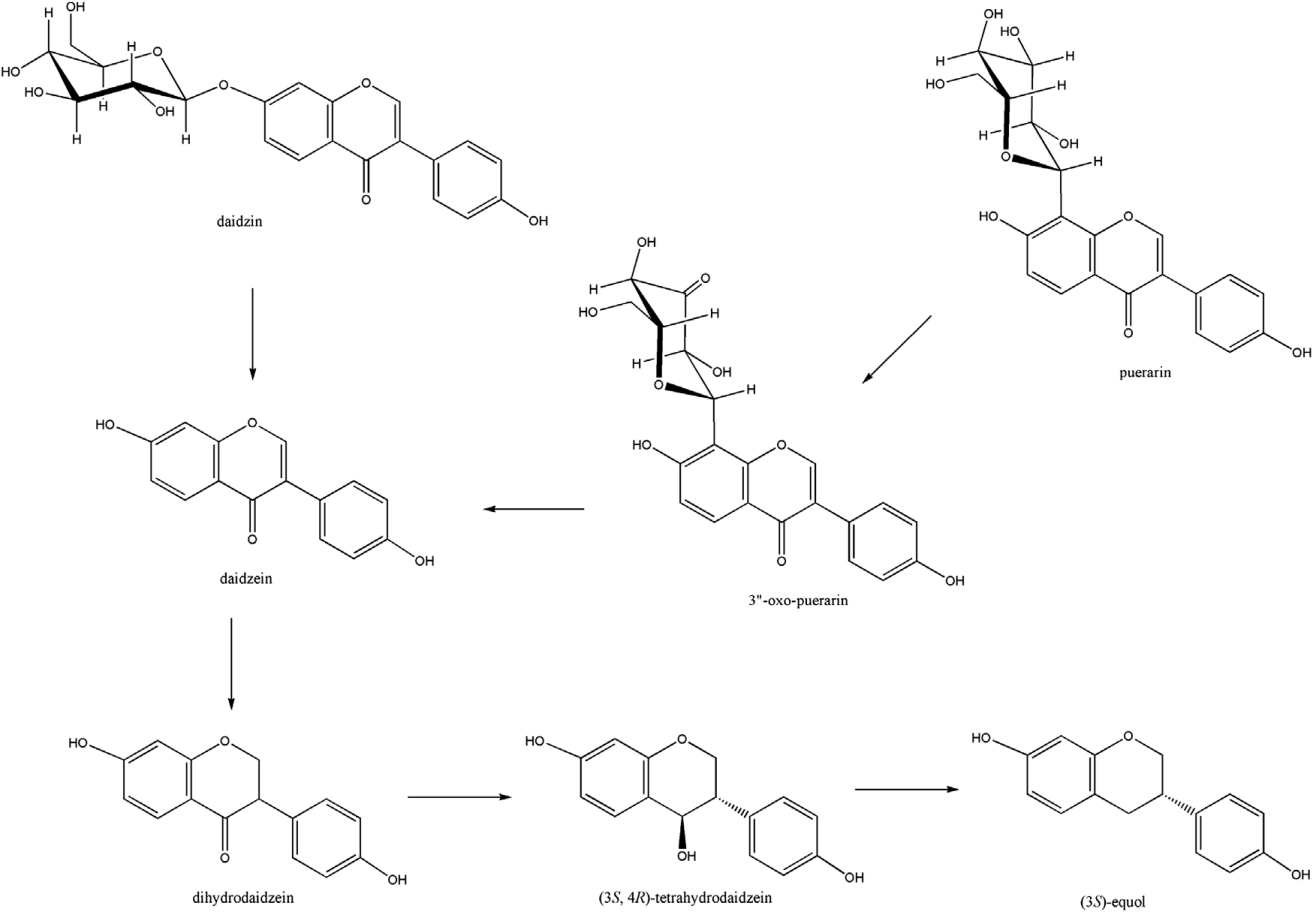
Isoflavone to *S*-equol metabolism in the human gut. Soy isoflavone daidzin is converted to daidzein by β-glucosidase, which is further reduced to (3*S*, 4*R*)-tetrahydrodaidzein through the (3*S*)-dihydrodaidzein. Puerarin was converted to daidzein through the formation of 3”-oxo-puerarin.

Puerarin is a major bioactive compound from the root of *Pueraria lobata* used in traditional medicine for the treatment of cardiovascular and cerebrovascular diseases, diabetes and diabetic complications, osteonecrosis, Parkinson’s disease, Alzheimer’s disease, endometriosis, and cancer (Zhou et al., 2014). It belongs to the isoflavone *C*-glycoside, and its stereospecific conversion to *S*-equol through the formation of daidzein in the human gut was elucidated (Kim et al., 2010; Park et al., 2011) (Figure 1). Gut metabolism of puerarin is known to begin with the regioselective oxidation of puerarin by NAD^+^-dependent oxidoreductase (Nakamura et al., 2020). Recently, 3”-oxidation of *C*-glycosides has been reported in environments such as soil and human gut, as a general biochemical metabolism of *C*-glycoside degradation (Kim et al., 2015; Kumano et al., 2021). However, how the regioselective oxidation of the C3 center of *C*-glycosides leads to the subsequent cleavage of the *C*-glycosidic bond is not well-understood yet.

**FIGURE 2:**
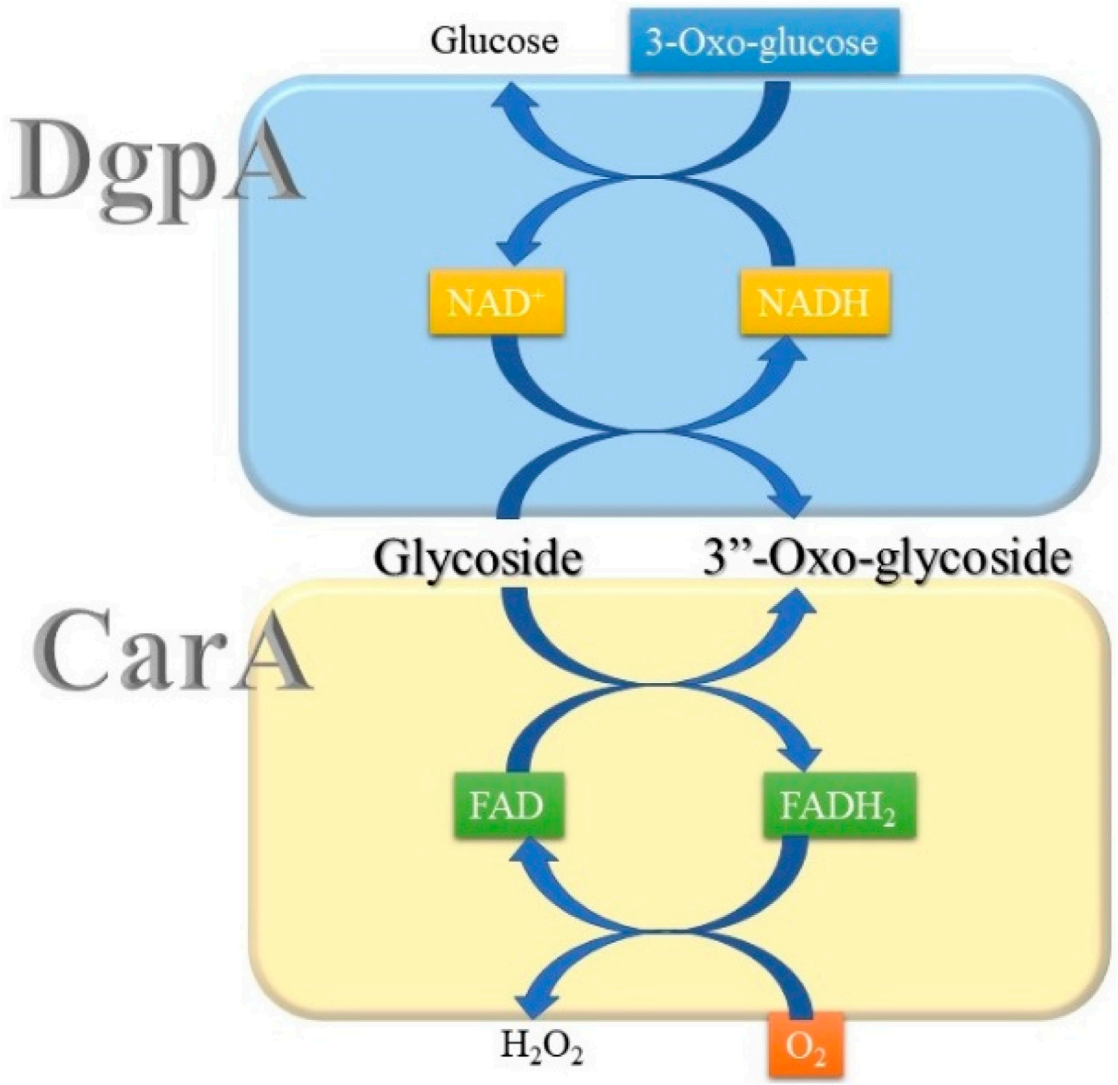
Cofactor regeneration by DgpA and CarA during the catalysis of *C*-glycoside oxidation.

Until now, two groups of oxidoreductases, GMC (glucose–methanol–choline) and Gfo/Idh/MocA families, have been reported to perform the regioselective oxidation of *C*-glycosides. Both families of enzymes, represented by CarA and DgpA, respectively, do not have sequence homology. While DgpA requires NAD^+^, CarA utilizes FAD for the oxidation of *C*-glycosides (Nakamura et al., 2020; Kumano et al., 2021). Accordingly, regeneration of the oxidized cofactors during catalysis is also different between these two oxidoreductases. In the case of DgpA, 3-oxo-glucose can oxidize NADH to regenerate NAD^+^. Molecular oxygen oxidizes FADH_2_ of CarA with the formation of hydrogen peroxide (Figure 2).

Recently, the convergent evolution of GMC and Gfo/Idh/MocA enzymes for metabolizing carbohydrates and *C*-/*O*-glycosides was suggested independently (Liu et al., 2007; Kuritani et al., 2020). The hypothesis implies that regiospecific oxidation of the monosaccharide moiety of the substrates is an important biochemical reaction in the biological system, though its significance is not completely understood. In contrast, *C*-glycoside metabolism by these enzymes provides an opportunity for the development of new biocatalysts due to the potential biotechnological applications of the reaction products. For example, it would allow the derivation of the sugar moiety of *C*-glycoside-derived antibiotics.

Due to the growing significance of glycoside oxidases in green biotechnology, the gene responsible for the oxidation of puerarin was cloned from the newly isolated *Dorea* sp. MRG-IFC3 strain (Mi et al., 2023). By utilizing the heterologously expressed enzyme D3dgpA, we have designed a more convenient biocatalytic system and studied the reactivity of D3dgpA with various glycosides and the effect of other monosaccharides. From the study, we have discovered that D3dgpA exhibited *O*-glycosidase activity for various *O*-glycosides as well as regiospecific glycoside oxidation. Furthermore, it was found by means of computational chemistry that the *O*-glycosidic bond cleavage observed from the reaction of D3dpgA is a non-enzymatic reaction due to the instability of 3”-oxo-*O*-glycoside products.

## 2 Materials and methods

### 2.1 Chemicals and reagents

All glycoside substrates used in this work were purchased from commercial sources and used without further purification (Supplementary Figure S1). The reagents for the enzymatic study were of biochemical grade, and all solvents used for HPLC analysis were of HPLC grade. Methyl β-D-3-oxo-glucopyranoside was synthesized and purified according to the published method (Tsuda et al., 1989). In detail, methyl β-D-glucopyranoside (500 mg) and 2 *eq* of (Bu_3_Sn)_2_O in chloroform (20 mL) were heated until the mixture was completely dissolved. The reaction mixture was cooled to 0°C, and 2 eq of bromine was added to the mixture with stirring. The color of the bromine disappeared in a few minutes, and the mixture was concentrated and poured into the silica gel column. The product was eluted with ethyl acetate and formed a white solid of methyl β-D-3-oxo-glucopyranoside (193 mg, 38% isolation yield) upon drying (Supplementary Figure S2). ^1^H NMR (600 MHz, methanol-*d*_4_) δ 4.28 (*d*, J = 6.0 Hz, C1-H), δ 4.23 (*d*, J = 6.0 Hz, C4-H), δ 4.12 (*d*, J = 6.0 Hz, C2-H), δ 3.94 (*dd*, J = 12, 1.8 Hz, C6-Ha), δ 3.80 (*dd*, J = 12, 6.0 Hz, C6-Hb), δ 3.58 (*s*, C1-OCH_3_), δ 3.51 (*m*, C5-H). ^13^C NMR (150 MHz, methanol-*d*_4_) δ 205.6, δ 105.3, δ 76.9, δ 76.8, δ 72.2, δ 61.1, δ 56.1. IR (KBr) 1735 cm^−1^(ν_C=O_).

7-*O*-Methylpuerarin was prepared by methylation of puerarin with methyl iodide. Puerarin (50 mg, 0.12 mmol) was dissolved in 1 mL DMF, and then K_2_CO_3_ (20 mg, 0.14 mmol) and methyl iodide in DMF (1/10; 75 μL, 0.12 mmol) were added. The mixture was stirred under a nitrogen atmosphere for 17 h at room temperature. After the disappearance of puerarin on TLC, the reaction mixture was acidified by acetic acid (0.5 mL), and the solvent was removed under vacuum. The product was purified by preparative TLC (chloroform: methanol=9:1), and a yellow solid (24.4 mg, 47% isolation yield) of 7-*O*-methylpuerarin (Supplementary Figure S3) was purified. *R*_*f*_ = 0.4 (silica gel TLC chloroform:methanol = 7:2). No fluorescence on 365 nm UV. UV; 250 nm, 305 nm. ^1^H NMR (600 MHz, DMF-*d*_7_): 3.4 (*s*; 3H; OCH_3_), 3.7–3.8 (*br s*, 3H); 4.1 (*m*, 1H); 4.4 (*m*, 1H); 5.17 (*br s*, 1H); 7.0 (*d*, J=8.4 Hz; 3H); 7.5 (*m*, 2H); 7.7 (*m*, 1H, H-5); 8.19 (*s*, 1H). ^13^C NMR (150 MHz, DMF-*d*_7_): δ 175.4, 162.2, 156.4, 155.9, 153.3, 130.4, 130.2, 127.0, 123.7, 119.0, 115.8, 115.3 110.1, 82.4, 79.7, 73.9, 71.9, 71.5, 62.6, 56.6.

### 2.2 Cloning and over-expression of D3dpgA

A polymerase chain reaction (PCR) was used to clone D3dgpA (GenBank number: OR238368.1). The genomic DNA of *Dorea* sp. MRG-IFC3 (KCTC25707) was used as a template. Two primers, 5’-gaa​ttc​aAT​GAG​TAA​ATT​AAA​AAT​TGG​TAT​TAT​TG-3’ (lower case letters are EcoRI site.) as a forward primer and 5’-gtc​gac​TTA​GAA​TTT​AAT​TGT​CTC​ATT​TGT​TT-3’ (lower case letters are SalI site) as a reverse primer were used. The PCR product was subcloned in pGEMT-easy vector (Promega) and sequenced. The D3dgpA was subcloned into the EcoRI/SalI site of the pET-duet 1 vector (Novagen). The resulting construct was transformed into *Escherichia coli* BL21 (DE3). To induce the protein, the overnight culture on LB plate was inoculated into LB broth containing 50 μg/mL of ampicillin and incubated at 37°C, 150 rpm until the OD at 600 nm reached 0.6. After adding IPTG to the final concentration of 0.5 mM, the culture was incubated at 18°C for 16 h. The culture was harvested by centrifugation at 5,000 g for 25 min at room temperature (yield 5 g/L of wet cells).

### 2.3 Purification of D3dgpA and enzyme assay

The overexpressed D3dgpA was purified using Ni-NTA affinity column chromatography. The cell pellet was suspended in the lysis buffer (1.0 g/mL, Tris 50 mM, NaCl 500 mM, pH 8.0) and subjected to sonication (15 s pulse/30 s pause, 36 cycles at 4°C). After centrifugation (25,000 g, 60 min, 4°C) of the cell lysate, the collected supernatant was filtered through a cellulose acetate filter (0.45 μm) and loaded on the Ni-NTA resin (Novagen). After thorough washing with 20 mM imidazole (Tris 50 mM, NaCl 500 mM, pH 8.0), step elution was performed. D3dgpA was eluted at a concentration between 250 mM and 400 mM of imidazole. Each fraction’s purity and quantification were measured by SDS-PAGE (Tris-glycine) and Bradford assay, respectively. Elution fractions were concentrated and dialyzed with MWCO 10KD UF (Spin-X® UF 20). After purification, the quantity of DgpA was determined by UV absorption at 280 nm or Bradford assay (595 nm).

All the enzyme reactions, including the study of enzyme kinetics and the effects of additives, were performed multiple times to check their reproducibility. A typical enzyme assay was performed with 700 equivalents of puerarin (10 mM in DMF) and methyl β-D-3-oxo-glucopyranoside (40 mM in water) with respect to D3dgpA in Tris buffer (10 mM, NaCl 10 mM, pH 8.0) at 37°C with stirring (250 rpm). The reaction was initiated by adding D3dgpA and stopped by adding ethyl acetate. The reaction product was extracted with ethyl acetate from twice the volume of the reaction mixture, which was dried under vacuum. The dried residue was dissolved in methanol. The methanolic solution was filtered with a syringe filter (0.22 μm, PTFE) before HPLC analysis. The reaction product of the assay was analyzed by Dionex Ultimate 3000 UHPLC with Kinetex® C18 column (1.7 μm, Phenomenex). The mobile phase was as follows: A, H_2_O with 0.1% acetic acid and B, acetonitrile. Linear gradient elution was adopted (5% of B at 0 min and increased to 55% at 15 min).

### 2.4 Activity of D3dgpA with alternative substrates

The reactivity of D3dgpA was monitored with various *C*-/*O*-Glycoside substrates (10 mM in DMF). To initiate the reaction, D3dgpA (10 μM) was added to the solution containing 1 mM of the glycoside substrate and methyl β-D-3-oxo-glucopyranoside in Tris buffer (10 mM, pH 8.0) at 37°C. The reaction mixture (100 μL) was stirred (250 rpm) for 30 min. The reaction was stopped by adding 200 μL of methanol, and the reaction product was filtered through the syringe filter (0.22 μm, PTFE) before HPLC analysis. The flow rate was 0.2 mL/min, the column temperature was 35°C, and the eluents of 1.0% formic acid and acetonitrile were used.

### 2.5 Kinetic study

D3dgpA (1 μg, 74 nM) was added to 5 mM HEPES buffer (pH 7.5) containing puerarin (76, 152, 304, 456, and 760 μM), methyl β-D-3-oxo-glucopyranoside (760 μM), and NaCl (5 mM), and the reaction mixture (300 μL) was incubated for 1 min at 36.5°C with stirring. The reaction was stopped by adding formic acid (15 μL). The reaction product was extracted by ethyl acetate (0.5 mL) twice, and the solution was subjected to dryness. Methanol (100 μL) was added to recover the products and analyzed by HPLC after filtration.

### 2.6 Effect of monosaccharides

Monosaccharides, including D-glucose, methyl β-glucopyranoside, arabinose, methyl β-galactopyranoside, levoglucosan, L-gulose, gluconolactone, methyl α-glucopyranoside, methyl α-mannose, and methyl β-galactose, were used to study the effect of 3”-oxo-puerarin formation on the D3dgpA activity. The reaction mixture (100 μL, pH 7.5, 5 mM HEPES) comprising 400 *equivalents* of puerarin and methyl β-D-3-oxo-glucopyranoside, and 800 *equivalents* of monosaccharide, with respect to D3dpgA, was reacted for 10 min (250 rpm, 36.5°C). The reaction was stopped by adding 5% formic acid. After centrifugation (18,000 rpm, 20 min), the supernatant was analyzed by HPLC. The relative inhibition was calculated from 3”-oxo-puerarin formation by comparing the activity of D3dgpA in the presence and absence of monosaccharide.

### 2.7 Analysis of the reaction product of methyl β-D-3-oxo-glucopyranoside

To confirm the function of β-D-3-oxo-glucopyranoside as an oxidant in the catalysis, the reaction product was analyzed after the enzyme reaction. The enzyme reaction mixture was filtered through a syringe filter and directly analyzed by LC/MS. UHPLC equipped with a Waters ACQUITY column (UPLC BEH C18 1.7 µm, 2.1 * 150 mm) was used for LC/MS analysis. The mobile phase comprising water 0.1% formic acid (v/v) (A) and acetonitrile 0.1% FA (v/v) (B) was used to apply the gradient system of B 1% (0 min), B 60% (6 min), and B 100% (7–8 min). The total run time was 10 min, and the flow rate was 0.4 mL/min at 50°C. A mass spectrometer equipped with an Orbitrap detector was used. Negative-mode MS in the range of 120–1000 m/z was applied for the analysis of carbohydrates.

### 2.8 Computational study

Computational chemistry was performed by Gaussian 16 packages combined with GaussView 6 (Gaussian, Inc. CT, United States). The bioenergetics of regiospecific puerarin oxidation by D3dgpA with 3-oxo-glucopyranose or methyl β-D-3-oxo-glucopyranoside were studied by comparing Gibbs free energy for each reaction in water. In detail, the most stable conformers of puerarin, 3”-oxo-puerarin, methyl β-D-3-oxo-glucopyranoside, β-D-3-oxo-glucopyranose, methyl β-D-glucopyranoside, and β-D-glucopyranose were found from the relaxed scan of dihedral angles. The free energy of the optimized structures was obtained at the B3BLYP level with the 6-311++G(d,p) basis set, including empirical dispersion and solvent effect.

The isomerization of 3”-oxo-puerarin to 2”-oxo-puerarin was investigated using the deprotonated anionic forms of both compounds. A transition state complex was obtained from the optimized compounds using the QST2 option. For the *O*-glycosidic bond cleavage of 3”-oxo-daidzin, a hydroxide ion was introduced, and the free energy of the optimized structure was compared with that of the 2”C-deprotonated 3”-oxo-daidzin and water.

## 3 Results

### 3.1 Characteristics of D3dgpA

The D3dgpA gene cloned from *Dorea* sp. MRG-IFC3 was of the same length as *dgpA* (Nakamura et al., 2020), but different in four nucleotides (OR238368.1). However, the primary structure of D3dgpA, comprising 367 amino acid residues, was identical to that of DgpA of strain PUE (BBG22493.1, Supplementary Figure S4). D3dgpA with His_6_-tag was purified by Ni-NTA affinity column chromatography and centrifugal filtration desalting. The estimated molecular weight of D3dgpA was 45 kD, and it seemed to form a hexamer in solution as reported (Supplementary Figure S5) (He et al., 2023). The catalysis by D3dgpA is known to follow the ping-pong mechanism. For example, the first reaction step produces 3”-oxo-glycoside and bound NADH, and the second reaction step reduces 3-oxo-glucose to regenerate NAD^+^ (Nakamura et al., 2020). In this study, the activity of D3dgpA was monitored with methyl β-D-3-oxo-glucopyranoside, instead of 3-oxo-glucose, because it is easy to prepare and not isomerized to the other isomers in solution. Like 3-oxo-glucose, methyl β-D-3-oxo-glucopyranoside acted as an oxidant to generate NAD^+^ during the catalysis. The reduced product, methyl β-D-glucopyranoside, was identified from the reaction mixture by LC-MS analysis (Supplementary Figure S6). However, other oxidants, such as glucuronolactone or α-ketoglutarate, reported to regenerate NAD^+^ in the same family of oxidoreductases (Taberman, et al., 2016), were not able to reconstitute the catalysis of D3dgpA.

The purified D3dgpA was found to contain an almost equimolar amount of NAD(H), and the ratio of NADH to NAD^+^ was determined to be 3 to 7 by HPLC analysis (Supplementary Figure S7). When additional amounts of NAD^+^ were introduced to the reaction mixture, NAD^+^ inhibited the activity of D3dgpA. Thus, allosteric regulation by NAD^+^ was suspected (Supplementary Figure S8).

D3dgpA showed the highest activity at pH 8.0 and exhibited high stability at room temperature (Supplementary Figures S9, S10). Approximately 80% of activity was maintained for 3 weeks. Although it was reported that manganese ions were required for the activity of D3dgpA in an earlier report (Nakamura et al., 2013), it was confirmed that D3dgpA does not require manganese or any other divalent metal ions, as evident from EDTA treatment.

In the presence of an excess amount of methyl β-D-3-oxo-glucopyranoside, D3dgpA showed Michaelis–Menten-type kinetics (Supplementary Figure S11). The kinetic parameters were obtained as K_M_ = 250 μM of puerarin, V_max_ = 140 μM/min, and *k*_cat_ = 32 s^−1^. The reported K_M_ value of DgpA was 330 μM (Nakamura et al., 2020). The catalytic efficiency of D3dgpA was 0.128 μM^−1^·s^−1^. The catalytic efficiencies of GMC family oxidoreductase enzymes, CarA and PsPOx, were reported as 0.226 μM^−1^·s^−1^ and 0.023 μM^−1^·s^−1^, respectively, for carminic acid and homoorientin (Kumano et al., 2021; Kostelac et al., 2024). Substrate inhibition of D3dgpA catalysis was observed at a concentration higher than 400 μM of puerarin. CarA also exhibited strong substrate inhibition at a concentration higher than 100 μM of carminic acid. When the same amounts of puerarin and daidzin were reacted with D3dgpA in the same reaction mixture, puerarin was converted faster than daidzin (Supplementary Figure S12).

**FIGURE 3:**
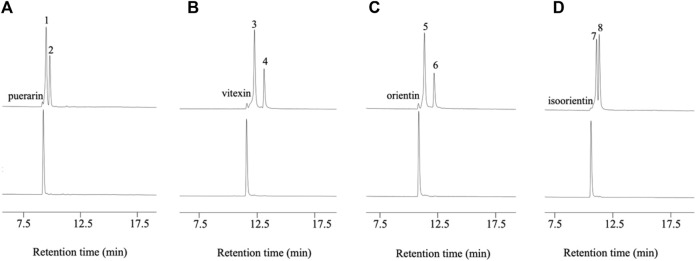
D3dgpA catalysis of C-glycosides in the presence (top) and absence of methyl β-D-3-oxo-glucopyranoside (bottom). **(A)** Puerarin was converted to 3”-oxo-puerarin (peak 1) and 2”-oxo-puerarin (peak 2), **(B)** vitexin was converted to 3”-oxo-vitexin (peak 3) and 2”-oxo-vitexin, **(C)** orientin was converted to 3”-oxo-orientin (peak 5) and 2”-oxo-orientin (peak 6), and **(D)** isoorientin was converted to 3”-oxo-isoorientin (peak 7) and 2”-oxo-isoorientin (peak 8).

D3dgpA did not catalyze the oxidation of puerarin in the absence of methyl β-D-3-oxo-glucopyranoside. However, in the presence of methyl β-D-3-oxo-glucopyranoside, it produced two products, 3”-oxo-puerarin (peak 1) and 2”-oxo-puerarin (peak 2) (Figure 3A) (Nakamura et al., 2020; Kumano et al., 2021). D3dgpA was also able to oxidize the other *C*-glycosides, including vitexin, orientin, and isoorientin, and 3”-oxo/2”-oxo-glycosides were produced at different ratios (Figure 3). These metabolites were also observed from the cell-free extract reaction of *Dorea* sp. MRG-IFC3, but in trace amounts. It is worthy of noting that *Dorea* sp. MRG-IFC3 cells cannot metabolize these *C*-glycosides (vitexin, orientin, and isoorientin) and only catalyzed the conversion of puerarin to daidzein and hexose enediolone (Mi et al., 2023). It was reported that CarA could not convert puerarin and orientin to the 3”-oxo-products (Kumano et al., 2021).

### 3.2 Reactivity of D3dgpA and the effects by monosaccharides

**FIGURE 4:**
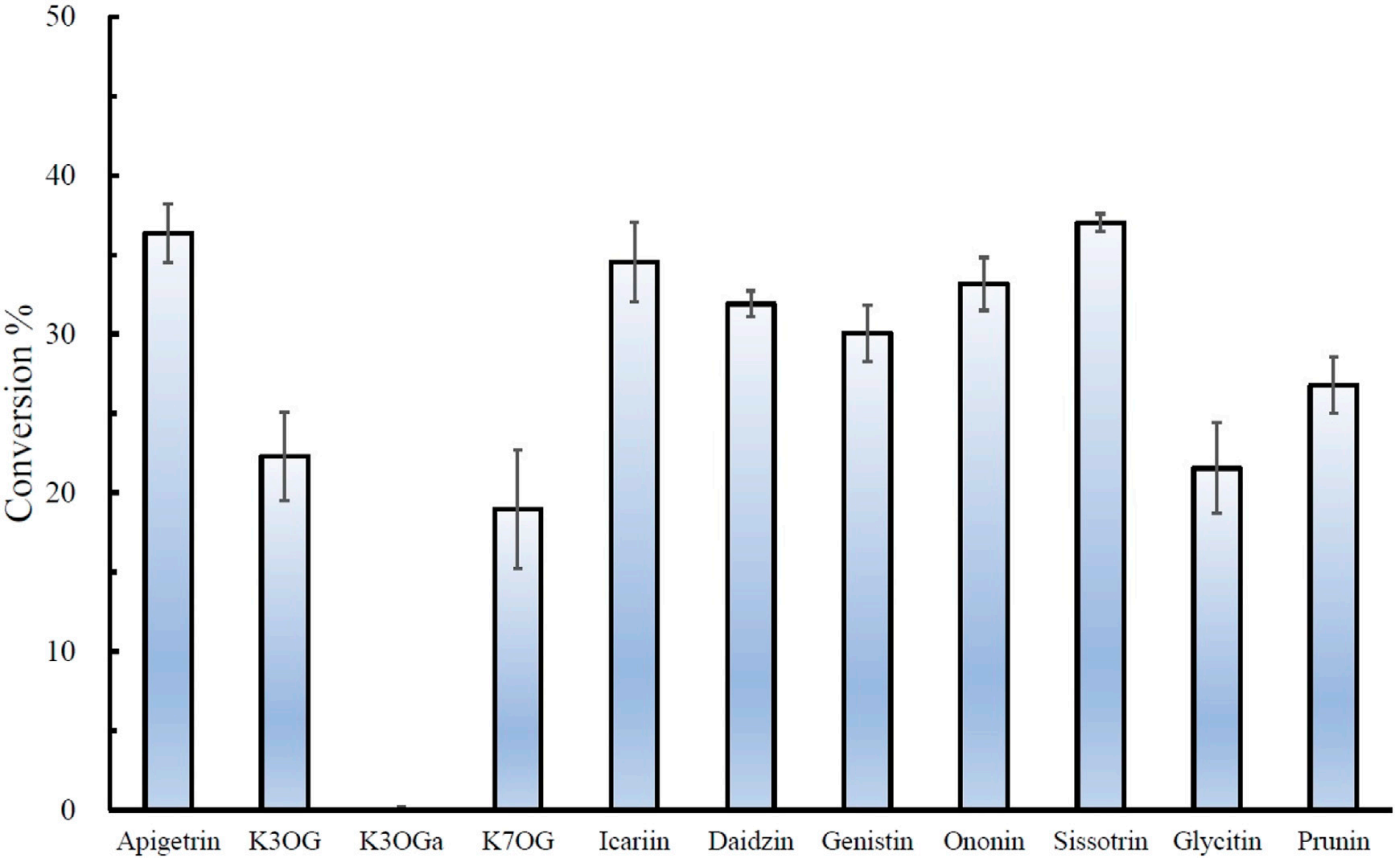
Relative conversion % of *O*-glycosides by D3dgpA. The reaction was run for 30 min, and the conversion ratio was obtained by measuring the disappearance of the substrate through HPLC analysis. K3OG, kaempferol 3-*O*-glycoside; K3OGa, kaempferol 3-*O*-galactoside; K7OG, kaempferol 7-*O*-glycoside.

The regiospecific oxidation of glycosides by Gfo/Idh/MocA and GMC family enzymes is recognized as a new universal pathway of natural glycoside degradation (Bitter et al., 2023; Kostelac et al., 2024). Therefore, various glycosides were reacted with D3dgpA to characterize the reactivity. D3dgpA catalyzed the oxidation of all *C*-glycosides, including 7-*O*-methylpuerarin (Table 1). However, no *C*-glycosidic bond cleavage was achieved by D3dpgA, as evident from the absence of aglycones. Instead, all 3”-oxo-*C*-glycoside products, including carminic acid, were isomerized to 2”-oxo-*C*-glycosides non-enzymatically (Figure 3). In the case of *O*-glycosides, 3”-oxo-*O*-glycosides were produced, which were further converted to aglycones (Figure 4; Supplementary Figure S13). The reactivity of D3dgpA with kaempferol glycosides was noticeable because no kaempferol production resulted from the reaction. D3dpgA produced 3”-oxo-kaempferol products from the reaction with kaempferol-3-*O*-glucoside and kaempferol-7-*O*-glucoside. D3dgpA did not react with kaempferol-7-*O*-galactoside. Icariin was converted by D3dgpA to the oxo-product. Since icariside II was produced, the 7-*O*-glucosyl unit of icariin seemed to be oxidized to 3”-oxo-icariin. Apigenin 7-*O*-glucuronide and diosmin were not converted by D3dgpA.

**TABLE 1:** Reactivity of D3dgpA.

Substrate		Product
*C*-glycosides	Puerarin	3”-Oxo-puerarin and 2”-oxo-puerarin
	Vitexin	3”-Oxo-vitexin and 2”-oxo-vitexin
	Orientin	3”-Oxo-orientin, and 2”-oxo- orientin
	Isoorientin	3”-Oxo-isoorientin and 2”-oxo- isoorientin
	Carminic acid	3”-Oxo-carminic acid and 2”-oxo-carminic acid
	7-*O*-methylpuerarin	3”-Oxo-7-*O*-methylpuerarin
*O*-glycosides	Daidzin	3”-Oxo-daidzin and daidzein
	Genistin	3”-Oxo-genistin and genistein
	Apigetrin	3”-Oxo-apigetrin and apigenin
	Ononin	3”-Oxo-ononin and formononetin
	Sissotrin	3”-Oxo-sissotrin and biochanin A
	Glycitin	3”-Oxo-glycitin and glycitein
	Kaempferol-3-*O*-glucoside	3”-Oxo-kaempferol-3-*O*-glucoside
	Kaempferol-3-O-galactoside	No reaction
	Kaempferol-7-*O*-glucoside	3”-Oxo-kaempferol-7-*O*-glucoside
	Icariin	Oxo-icariin, icariside II
Other glycosides	Bergenin	Oxo-bergenin and derivatives
	Apigenin-7-*O*-glucuronide	No reaction
	Diosmin	No reaction
	Amygdalin	Oxo-amygdalin
	Naringenin 7-*O*-glucoside	3”-Oxo-naringenin 7-*O*-glucoside and naringenin

From the reactivity study of D3dgpA, *C*-glycosides were converted to 3”-oxo-and 2”-oxo-products, and the latter were formed by non-enzymatic reaction as observed from DgpA and CarA (Bitter et al., 2023). Most *O*-glycosides, except kaempferol *O*-glycosides, were converted to 3”-oxo-products and aglycones. It appears that the formation of aglycones depends on the stability of 3”-oxo-*O*-glycosides. In addition, the regiospecific oxidation by D3dgpA was specific to the glucose group attached to the aglycones, and no reaction was observed for the glycosides with galactose, rhamnose, and glucuronide.

**FIGURE 5:**
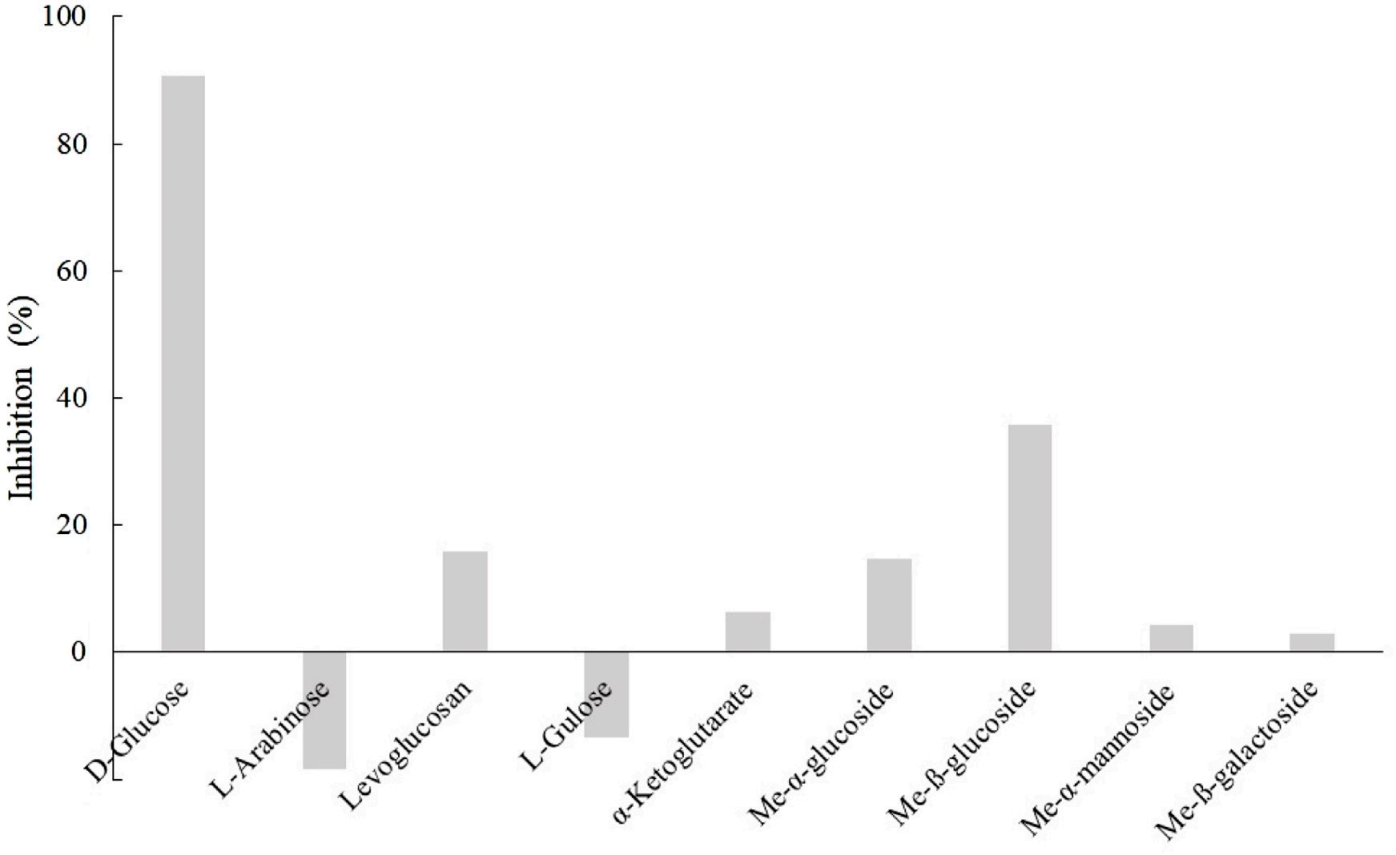
Monosaccharide inhibition on puerarin oxidation by D3dgpA.

Because D3dgpA reacted with various glycosides by acting on the glycosyl group, the effect of other monosaccharides on puerarin oxidation was investigated to evaluate whether they interfered with puerarin oxidation by D3dgpA (Figure 5). Among the tested monosaccharides, D-glucose exhibited the most inhibitory effects on the oxidation of puerarin in the presence of methyl β-D-3-oxo-glucopyranoside. The inhibitory effect of monosaccharides decreased in the order of D-glucose > methyl *ß*-glucoside > methyl α-glucoside ≈ levoglucosan. Arabinose and gulose appeared to potentiate the activity of D3dgpA for the oxidation of puerarin. The inhibition by D-glucose emphasizes high substrate specificity for glucosides and can be considered a product inhibition since D3dgpA catalyzes an equilibrium reaction *vide infra* (Choi et al., 2023). The results can also be applied to the biotechnological application of *Dorea* sp. MRG-IFC3 when the medium composition is optimized.

### 3.3 Bioenergetics and reaction mechanism of D3dgpA catalysis

One of the rationales for the investigation of biochemical metabolism is bioenergetics of the enzyme-catalyzed reaction. With 3-oxo-glucose, the reaction of 3”-oxo-puerarin formation catalyzed by D3dgpA was found to have an equilibrium constant close to 1 (Eq. 1) (Choi et al., 2023). Since methyl β-D-3-oxo-glucopyranoside was used for the study of D3dgpA, we have first compared the free energy change of the D3dgpA-catalyzed reaction in the presence of methyl β-D-3-oxo-glucopyranoside (Eq. 2) by means of DFT computational chemistry at the level of G6-311++(d,p). The reaction was found to be endergonic by 0.27 kcal/mol under the same conditions. Although the reaction became slightly more endergonic than the reaction utilizing 3-oxo-glucose by 0.09 kcal/mol, the equilibrium constant was practically the same as shown in Eq. 2. Therefore, methyl β-D-3-oxo-glucopyranoside was found to be a suitable alternative oxidant for D3dgpA from the perspective of bioenergetics.

puerarin+3−oxo−glucose ↔ 3”−oxo−puerarin+glucose; 0.18 kcal/mol (1)

puerarin+methyl β−D−3−oxo−glucopyranoside ↔ 3”−oxo−puerarin+methyl β−D−glucopyranoside; 0.27 kcal/mol. (2)

Likewise, D3dgpA-catalyzed oxidation of daidzin was found to be slightly endergonic (Eq. 3). The formation of 3”-oxo-daidzin by D3dgpA appeared to be unfavorable thermodynamically, by 0.70 kcal/mol, when compared to the formation of 3”-oxo-puerarin (Eq. 1). It could be one of the reasons why D3dgpA is more reactive against puerarin than daidzin (Supplementary Figure S12).

daidzin+3−oxo−glucose ↔ 3”−oxo−daidzin+glucose; 0.88 kcal/mol. (3)

Next, *O*-glycosidic cleavage reaction of 3”-oxo-*O*-glycosides was investigated with daidzin. After the formation of 3”-oxo-daidzin by D3dgpA, the product was converted to daidzein and hexose enediolone. Previously, the cleavage of *O*-glycosidic bond of 3”-oxo-*O*-glycosides was proposed as a non-enzymatic reaction (Kumano et al., 2021). To check the proposal, we have investigated the thermochemistry of the reaction. Whereas the *C*-glycosidic bond cleavage of 3”-oxo-puerarin is slightly endergonic (Eq. 4), the conversion of 3”-oxo-daidzin to daidzein and hexose enediolone was highly exergonic, by 10.13 kcal/mol (Eq. 5). Therefore, it was found that the cleavage of 3”-oxo-daidzin *O*-glycosidic bond was favored thermodynamically. To our surprise, 3”-oxo-daidzin was converted to daidzein and hexose enediolone during the geometry optimization, when the hydroxide ion was introduced. Thus, the reaction appears to happen spontaneously in the presence of a hydroxide ion.

3”−oxo−puerarin ↔ daidzein+hexose enediolone; 0.22kcal/mol. (4)

3”−oxo−daidzin → daidzein+hexose enediolone; −10.13kcal/mol. (5)

Finally, the isomerization of 3”-oxo-*C*-glycosides was investigated with 3”-oxo-puerarin. The free energy difference between 3”-oxo-puerarin and 2”-oxo-puerarin was 2.82 kcal/mol, which is equivalent to 99:1 distribution in aqueous environments. Such a distribution of 3”-oxo-puerarin and 2”-oxo-puerarin was indeed reported by NMR measurement (Nakamura, et al., 2019).

**FIGURE 6:**
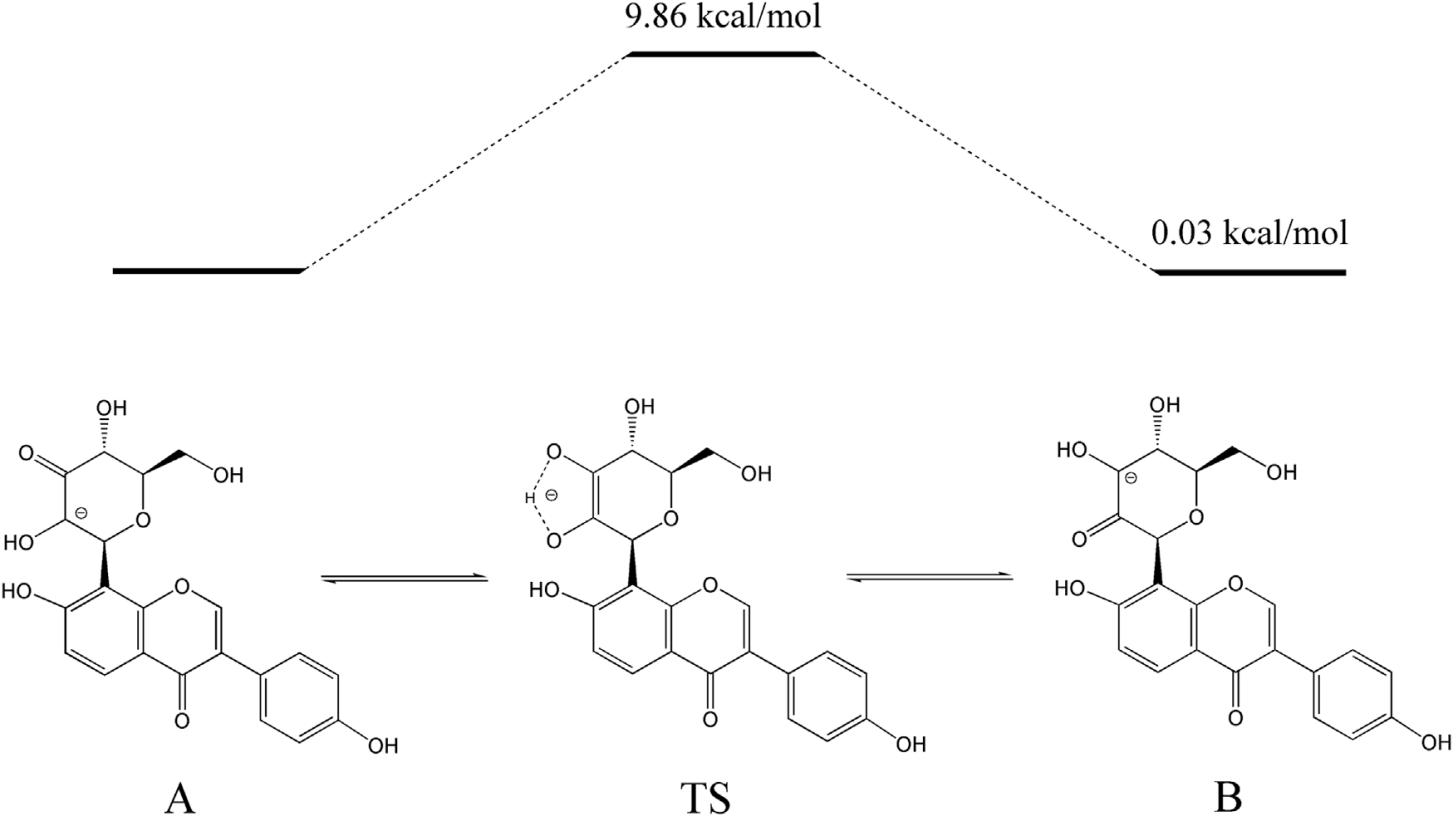
Proposed pathway for the isomerization of 3”-oxo-puerarin to 2”-oxo-puerarin **(B)**. Intramolecular hydrogen transfer between two deprotonated 3”-oxo-puerarin anion **(A)** and 2”-oxo-puerarin anion **(B)** had a transition state complex (TS) in a higher energy state by 9.86 kcal/mol.

The activation energy of the isomerization between 3”-oxo-puerarin and 2”-oxo-puerarin was also calculated by transition state search (Figure 6). The 2”C-deprotonated species of 3”-oxo-puerarin (A) and the 3”C-deprotonated species of 2”-oxo-puerarin (B) were almost the same in free energy. Enediolate anion species (TS) was found as a transition state complex, whose free energy was higher than that of the 3”-oxo-puerarin anion (A) only by 9.86 kcal/mol. Therefore, the isomerization of 3”-oxo-*C*-glycoside to 2”-oxo-*C*-glycoside was found feasible thermodynamically.

**FIGURE 7:**
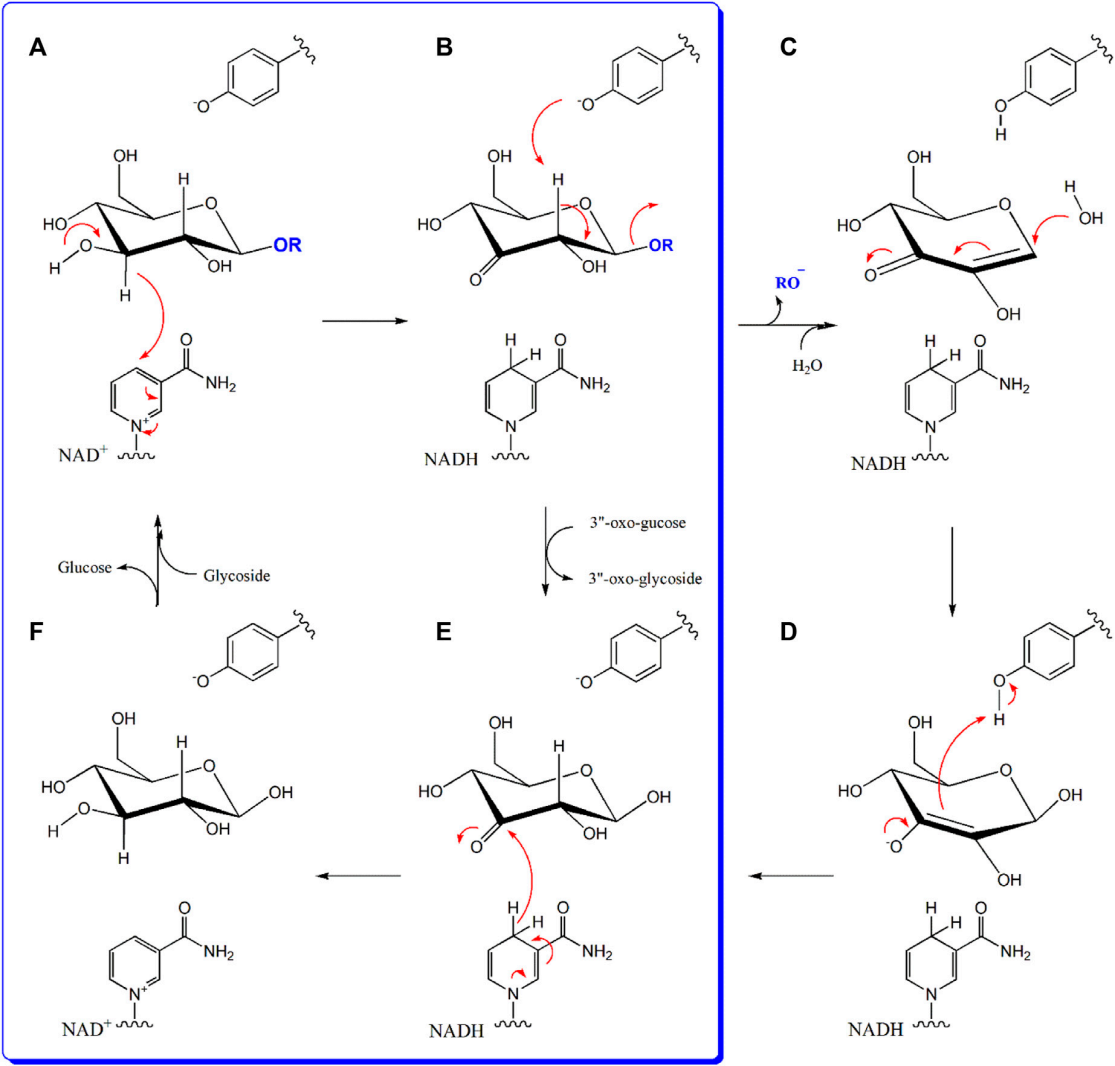
The reaction mechanism of D3dgpA in comparison to other glycoside oxidoreductases. D3dgpA-catalyzed reaction pathway is shown in the blue panel. General base deprotonates the 3”O-H and the 3”-hydride is subsequently transferred to NAD^+^ **(A)**. In the case of other glycoside hydrolases, tyrosyl residue in the active site deprotonates the 2”-H to break the O-glycosidic bond with the formation of hexose enediolone **(B)**. A water molecule is added on the β-position of hexose enediolone **(C)** and protonation of C3 position is achieved by the tyrosyl residue to form the 3-oxo-glucose **(D)**. However, D3dgpA releases the 3”-oxo-product and binds 3-oxo-glucose as the second substrate, to regenerate NAD^+^ **(E)**. With the formation of NAD^+^, the formed glucose molecule leaves the active site and the next glycoside binds to initiate the next catalytic cycle **(F)**.

The reaction catalyzed by D3dgpA is reminiscent of other Gfo/Idh/MocA-like oxidoreductases, which also utilize NAD(P)^+^ to form glycoside oxo-products. However, D3dgpA from *Dorea* sp. MRG-IFC3 is unique, in that it reacts with *C*-glycosides and produces 3”-oxo-products instead of the hydrolyzed products. D3dgpA appears to follow the same reaction mechanism as other enzymes utilizing NAD^+^ for the biochemical oxidation of 3”C-OH of the glycosyl group (Figure 7). However, other oxidoreductases go through the subsequent deprotonation of the acidic 2”C-H by the tyrosyl anion residue, which facilitates the cleavage of C-O-glycosidic bond to release the aglycone (Figure 7B). Then, the water is added to the hexose enediolone intermediate to yield the enolate intermediate (Figure 7C). The second intermediate is protonated by the tyrosyl residue to produce 3-oxo-glucose (Figure 7D). The third intermediate, 3-oxo-glucose, is then reduced to glucose by NADH, to regenerate NAD^+^ (Figure 7E). Finally, the glucose is exchanged with the new glycoside for the next cycle of reaction (Figure 7F). In the case of D3dgpA, the catalysis cannot afford the cleavage of the *C*-glycosidic bond of 3”-oxo-*C*-glycoside even after 2”C-H deprotonation (Figure 7B). Thus, it should release 3”-oxo-*C*-glycoside from the catalytic site and accept 3-oxo-glucose to regenerate NADH (Figure 7E). Namely, it catalyzes the reaction steps of A → B → E → F → A in Figure 7. Compared to other Gfo/Idh/MocA and GMC family enzymes, D3dgpA only catalyzed regioselective oxidation of *C*-/*O*-glycosides and did not undergo further hydration and regeneration of cofactors (Figure 7). A recent report on the GMC family enzyme, GlycDH, also emphasized that the substrate specificity and *C*-/*O*-glycosidic bond cleavage of the related enzymes cannot be explained by the active site structure or the Mn^2+^ cofactor (Bitter et al., 2023).

## 4 Discussion

In this report, we have studied the characteristics and reactivity of D3dgpA, a Gfo/Idh/MocA family glycoside oxidoreductase. Expression of D3dgpA in *Dorea* sp. MRG-IFC3 was induced by puerarin (Mi et al., 2023), and it appeared to form a hexameric quaternary structure. Though D3dgpA exhibited higher reactivity for *C*-glycosides than *O*-glycosides, it also efficiently catalyzed the oxidation of *O*-glycosides (Table 1, Figure 4). One of the reasons we specifically investigated the activity of D3dgpA was the high substrate specificity of *Dorea* sp. MRG-IFC3 in the cleavage of the *C*-glycosidic bond. The IFC3 strain only metabolized puerarin, but not the other *C*-glycosides. From the reactivity study of D3dgpA, it is now clear that puerarin-specific *C*-deglycosylation is due to the *C*-deglycosidase, D3dgpBC, which metabolizes 3”-oxo-puerarin (Figure 1).

D3dgpA catalyzed the oxidation of glycosides, resulting in more than a single product, but it is believed that the direct product formed from the catalysis is 3”-oxo-glycosides. In the case of *C*-glycosides, 3”-oxo-*C*-glycosides were isomerized to 2”-oxo-*C*-glycosides through keto-enol tautomerization. The ratio of 3”-oxo- to 2”-oxo-*C*-glycoside was different depending on *C*-glycoside products because of the thermodynamic stability of the isomers produced by the non-enzymatic reaction. From the computational chemistry approach, it appeared that the activation energy of the isomerization between 3”-oxo and 2”-oxo-*C*-glycosides was low enough to occur non-enzymatically. In addition, 3”-oxo-*O*-glycosides products were further converted to 2”-oxo-*O*-glycosides and cleavage products of the *O*-glycosidic bond, aglycone and hexose enediolone (Figure 7D), through the non-enzymatic reaction. Therefore, 2”-oxo-*C*-glycosides, 2”-oxo-*O*-glycosides, aglycones, and hexose enediolone are the products formed by the non-enzymatic reactions due to the stability of 3”-oxo-glycosides. The results also imply a significant energy barrier for cleavage of the *C*-glycosidic bond of 3”-oxo-glycosides, which cannot be overcome with a Gfo/Idh/MocA family glycoside oxidoreductase. The cleavage of the *C*-glycosidic bond of 3”-oxo-*C*-glycosides is catalyzed only by *C*-deglycosidase (Mori et al., 2021; Choi et al., 2023).

Along with the structural features, the similarity of the proposed mechanism between *O*- and *C*-glycosidic bond cleavages observed from 3”-oxo-glycosides still puzzles scientists as to why the C-glycosidic bond cleavage is feasible only by *C*-deglycosidase (Bitter et al., 2023). The Mn^2+^ ion in the active site of *C*-deglycosidase and/or the α-OH group of the aglycone adjacent to the 3”-oxo-glucosyl unit of the substrate is not sufficient to guarantee the cleavage of the *C*-glycosidic bond. Otherwise, glycoside hydrolase family 4 enzymes, belonging to the Gfo/Idh/MocA family, could cleave the *C*-glycosidic bond of 3”-oxo-*C*-glycosides (Varrot et al., 2005). As found from our computational study, 3”-oxo-*C*-glycosides are much more stable than 3”-oxo-*O*-glycosides. Therefore, it appears the other catalytic mechanisms are required to lower the activation energy of the *C*-glycosidic bond cleavage and the Mn^2+^ ion in the active site and structural features of the substrate.

Whereas a plethora of glycoside-metabolizing biochemical reactions, including lyase, hydrolase, and transferase, are available, redox-active cofactor-dependent glycoside oxidoreductases, such as Gfo/Idh/MocA and GMC family enzymes, have emerged as a new group of glycoside-metabolizing enzymes (Bitter et al., 2023; Taborda et al., 2023). These glycoside oxidoreductases require NAD(P)^+^ or flavin for the oxidation of glycosides. Whereas flavin-utilizing enzymes utilize molecular oxygen and other abiological oxidants, such as 1,4-benzoquinone, for the catalysis (Figure 2), NAD(P)^+^-dependent enzymes regenerate the oxidant cofactor by reducing other metabolites, such as 3-oxo-glucose and α-ketoglutarate. The emerging mechanism of glycoside catabolism appears to involve the formation of 3”-oxo-glycosides, but their utilization in biochemistry is not limited to the degradation of glycosides to aglycones. These enzymes can also be used for biotechnological applications to provide new bioactive *C*-glycosides. Especially, D3dgpA, stable at room temperature for 3 weeks, appears to have by far the largest substrate binding site among the reported Gfo/Idh/MocA family enzymes (Yip et al., 2004; Varrot et al., 2005; Thoden and Holden, 2010; Thoden and Holden, 2011; Mukherjee et al., 2019; Kuritani et al., 2020; Yoshiwara et al., 2020; Kumano et al., 2021). Hence, D3dgpA can be used for the regioselective functionalization of the known bioactive *C*-glycosides through the 3”-oxo-*C*-glycosides (Kitamura et al., 2018). Often, regioselective chemical functional group transformation using carbohydrates is very difficult, but D3dgpA provides the new enzyme synthesis.

## 5 Conclusion

In this report, we have investigated the reactivity of D3dgpA, cloned from *Dorea* sp. MRG-IFC3. D3dgpA has exhibited a broad substrate spectrum for *C*-/*O*-glycosides. Regiospecific oxidation of the glucosyl unit of the glycosides resulted in the formation of 3”-oxo-*C*-/*O*-glycosides. The formation of 2”-oxo-*C*-glycosides was achieved by non-enzymatic isomerization in the reaction mixture. In addition, 3”-oxo-*O*-glycosides were also converted to aglycones abiologically due to the low transition state and instability of 3”-oxo-*O*-glycosides. Our study has made D3dgpA a potentially important enzyme for valorizing natural glycosides.

## Data Availability

The original contributions presented in the study are included in the article/Supplementary Material; further inquiries can be directed to the corresponding author.

## References

[B1] Bitter J. Pfeiffer M. Borg A. J. E. Kuhlmann K. Pavkov-Keller T. Sánchez-Murcia P. A. (2023). Enzymatic *β*-elimination in natural product *O*- and *C*-glycoside deglycosylation. Nat. Commun. 14, 7123. 10.1038/s41467-023-42750-0 37932298 PMC10628242

[B2] Choi J. Kim Y. Eser B. E. Han J. (2023). Theoretical study on the glycosidic C–C bond cleavage of 3’’-oxo-puerarin. Sci. Rep. 13, 16282. 10.1038/s41598-023-43379-1 37770535 PMC10539306

[B3] Chong Y. Kim B. G. Park Y. J. Yang Y. Lee S. W. Lee Y. (2023). Production of four flavonoid *C*-glucosides in *Escherichia coli* . J. Agric. Food Chem. 71 (13), 5302–5313. 10.1021/acs.jafc.3c00297 36952620

[B4] He P. Wang S. Li S. Liu S. Zhou S. Wang J. (2023). Structural mechanism of a dual-functional enzyme DgpA/B/C as both a *C*-glycoside cleaving enzyme and an *O*- to *C*-glycoside isomerase. Acta Pharm. Sin. B 13 (1), 246–255. 10.1016/j.apsb.2022.05.022 36815035 PMC9939296

[B5] Kim E. M. Seo J. H. Baek K. Kim B. G. (2015). Characterization of two-step deglycosylation via oxidation by glycoside oxidoreductase and defining their subfamily. Sci. Rep. 5, 10877. 10.1038/srep10877 26057169 PMC4650693

[B6] Kim M. Marsh E. N. G. Kim S. U. Han J. (2010). Conversion of (3*S*,4*R*)-tetrahydrodaidzein to (3*S*)-equol by THD reductase: proposed mechanism involving a radical intermediate. Biochemistry 49 (26), 5582–5587. 10.1021/bi100465y 20515029

[B7] Kitamura K. Ando Y. Matsumoto T. Suzuki K. (2018). Total synthesis of aryl *C*-glycoside natural products: strategies and tactics. Chem. Rev. 118 (4), 1495–1598. 10.1021/acs.chemrev.7b00380 29281269

[B8] Kostelac A. Taborda A. Martins L. O. Haltrich D. (2024). Evolution and separation of actinobacterial pyranose and C-glycoside-3-oxidases. Appl. Environ. Microbiol. 90, e0167623. 10.1128/aem.01676-23 38179968 PMC10807413

[B9] Kumano T. Hori S. Watanabe S. Terashita Y. Yu H. Y. Hashimoto Y. (2021). FAD-dependent *C*-glycoside–metabolizing enzymes in microorganisms: screening, characterization, and crystal structure analysis. Proc. Natl. Acad. Sci. 118 (40), e2106580118. 10.1073/pnas.2106580118 34583991 PMC8501837

[B10] Kuritani Y. Sato K. Dohra H. Umemura S. Kitaoka M. Fushinobu S. (2020). Conversion of levoglucosan into glucose by the coordination of four enzymes through oxidation, elimination, hydration, and reduction. Sci. Rep. 10 (1), 20066. 10.1038/s41598-020-77133-8 33208778 PMC7676230

[B11] Liu Q. P. Sulzenbacher G. Yuan H. Bennett E. P. Pietz G. Saunders K. (2007). Bacterial glycosidases for the production of universal red blood cells. Nat. Biotechnol. 25 (4), 454–464. 10.1038/nbt1298 17401360

[B12] Mi H. T. N. Chaiyasarn S. Kim H. Han J. (2023). *C*-Glycoside-metabolizing human gut bacterium, *Dorea* sp. MRG-IFC. J. Microbiol. Biotechnol. 33 (12), 1606–1614. 10.4014/jmb.2308.08021 37789701 PMC10772555

[B13] Min S. Y. Park C. H. Yu H. W. Park Y. J. (2021). Anti-inflammatory and anti-allergic effects of saponarin and its impact on signaling pathways of RAW 264.7, RBL-2H3, and HaCaT Cells. Int. J. Mol. Sci. 22 (16), 8431. 10.3390/ijms22168431 34445132 PMC8395081

[B14] Mori T. Kumano T. He H. Watanabe S. Senda M. Moriya T. (2021). C-Glycoside metabolism in the gut and in nature: identification, characterization, structural analyses and distribution of CC bond-cleaving enzymes. Nat. Comm. 12 (1), 6294. 10.1038/s41467-021-26585-1 PMC8563793 34728636

[B15] Mukherjee K. Huddleston J. P. Narindoshvili T. Nemmara V. V. Raushel F. M. (2019). Functional characterization of the ycjQRS gene cluster from *Escherichia coli*: a novel pathway for the transformation of D-gulosides to D-glucosides. Biochemistry 58 (10), 1388–1399. 10.1021/acs.biochem.8b01278 30742415 PMC6613369

[B16] Nakamura K. Komatsu K. Hattori M. Iwashima M. (2013). Enzymatic cleavage of the *C*-glucosidic bond of puerarin by three proteins, Mn^2+^, and oxidized form of nicotinamide adenine dinucleotide. Biol. Pharm. Bull. 36 (4), 635–640. 10.1248/bpb.b12-01011 23328408

[B17] Nakamura K. Zhu S. Komatsu K. Hattori M. Iwashima M. (2019). Expression and characterization of the human intestinal bacterial enzyme which cleaves the *C*-glycosidic bond in 3”-oxo-puerarin. Biol. Pharm. Bull. 42 (3), 417–423. 10.1248/bpb.b18-00729 30626800

[B18] Nakamura K. Zhu S. Komatsu K. Hattori M. Iwashima M. (2020). Deglycosylation of the isoflavone *C*-glucoside puerarin by a combination of two recombinant bacterial enzymes and 3-oxo-glucose. Appl. Environ. Microbiol. 86 (14), e00607–e00620. 10.1128/AEM.00607-20 32385077 PMC7357486

[B19] Ni R. Liu X. Y. Zhang J. Z. Fu J. Tan H. Zhu T. T. (2022). Identification of a flavonoid *C*-glycosyltransferase from fern species *Stenoloma chusanum* and the application in synthesizing flavonoid *C*-glycosides in *Escherichia coli* . Microb. Cell Factories 21 (1), 210–217. 10.1186/s12934-022-01940-z PMC9563126 36242071

[B20] Park H. Y. Kim M. Han J. (2011). Stereospecific microbial production of isoflavanones from isoflavones and isoflavone glucosides. Appl. Microbiol. Biotech. 91 (4), 1173–1181. 10.1007/s00253-011-3310-7 21562980

[B21] Peng Y. Gan R. Li H. Yang M. McClements D. J. Gao R. (2021). Absorption, metabolism, and bioactivity of vitexin: recent advances in understanding the efficacy of an important nutraceutical. Crit. Rev. Food Sci. Nutr. 61 (6), 1049–1064. 10.1080/10408398.2020.1753165 32292045

[B22] Taberman H. Parkkinen T. Rouvinen J. (2016). Structural and functional features of the NAD(P) dependent Gfo/Idh/MocA protein family oxidoreductases. Protein. Sci. 25 (4), 778–786. 10.1002/pro.2877 26749496 PMC4941211

[B23] Taborda A. Frazão T. Rodrigues M. V. Fernández-Luengo X. Sancho F. Lucas M. F. (2023). Mechanistic insights into glycoside 3-oxidases involved in C-glycoside metabolism in soil microorganisms. Nat. Commun. 14, 7289. 10.1038/s41467-023-42000-3 37963862 PMC10646112

[B24] Tao M. Li R. Zhang Z. Wu T. Xu T. Zogona D. (2022). Vitexin and isovitexin act through inhibition of insulin receptor to promote longevity and fitness in *Caenorhabditis elegans* . Mol. Nutr. Food Res. 66 (17), 2100845. 10.1002/mnfr.202100845 35413150

[B25] Thoden J. B. Holden H. M. (2010). Structural and functional studies of WlbA: a dehydrogenase involved in the biosynthesis of 2, 3-diacetamido-2, 3-dideoxy-D-mannuronic acid. Biochemistry 49 (36), 7939–7948. 10.1021/bi101103s 20690587 PMC4241754

[B26] Thoden J. B. Holden H. M. (2011). Biochemical and structural characterization of WlbA from *Bordetella pertussis* and *Chromobacterium violaceum*: enzymes required for the biosynthesis of 2, 3-diacetamido-2, 3-dideoxy-D-mannuronic acid. Biochemistry 50 (9), 1483–1491. 10.1021/bi101871f 21241053 PMC3050068

[B27] Tsuda Y. Hanajima M. Matsuhira N. Okuno Y. Kanemitsu K. (1989). Regioselective mono-oxidation of non-protected carbohydrates by brominolysis of the tin intermediates. Biol. Pharm. Bull. 37 (9), 2344–2350. 10.1248/cpb.37.2344

[B28] Varrot A. Yip V. L. Li Y. Rajan S. S. Yang X. Anderson W. F. (2005). NAD^+^ and metal-ion dependent hydrolysis by family 4 glycosidases: structural insight into specificity for phospho-β-D-glucosides. J. Mol. Biol. 346 (2), 423–435. 10.1016/j.jmb.2004.11.058 15670594

[B29] Xiao J. Capanoglu E. Jassbi A. R. Miron A. (2016). Advance on the flavonoid *C*-glycosides and health benefits. Crit. Rev. Food Sci. Nutr. 56 (1), S29–S45. 10.1080/10408398.2015.1067595 26462718

[B30] Xie L. Deng Z. Zhang J. Dong H. Wang W. Xing B. (2022). Comparison of flavonoid *O*-glycoside, *C*-glycoside and their aglycones on antioxidant capacity and metabolism during *in vitro* digestion and *in vivo* . Foods 11 (6), 882. 10.3390/foods11060882 35327304 PMC8949116

[B31] Yip V. L. Varrot A. Davies G. J. Rajan S. S. Yang X. Thompson J. (2004). An unusual mechanism of glycoside hydrolysis involving redox and elimination steps by a family 4 β-glycosidase from *Thermotoga maritima* . J. Am. Chem. Soc. 126 (27), 8354–8355. 10.1021/ja047632w 15237973

[B32] Yoshiwara K. Watanabe S. Watanabe Y. (2020). Crystal structure of bacterial L-arabinose 1-dehydrogenase in complex with L-arabinose and NADP^+^ . Biochem. Biophys. Res. Comm. 530 (1), 203–208. 10.1016/j.bbrc.2020.07.071 32828286

[B33] Zhou Y. X. Zhang H. Peng C. (2014). Puerarin: a review of pharmacological effects. Phytother. Res. 28 (7), 961–975. 10.1002/ptr.5083 24339367

