# Supplementary material for: Glycoside-metabolizing oxidoreductase D3dgpA from human gut bacterium
Source: Front Bioeng Biotechnol. 2024 Jun 28;12:1413854. doi: 10.3389/fbioe.2024.1413854 (PMC11239390; doi:10.3389/fbioe.2024.1413854)

Supplementary Materials to

Glycoside-Metabolizing Oxidoreductase D3dgpA from Human gut bacterium

Heji Kim^1^, Huynh Thi Ngoc Mi^1^, Joong-Hoon Ahn^2^, Jong Suk Lee^3^, Bekir Engin Eser^4^, Jongkeun Choi^5^, Jaehong Han^1*^

Table S1. Human gut bacteria reported to metabolize C-glycosides.

| **Strain** | **Substrate** | **Metabolite** | **Activity** | **Reference** |
| --- | --- | --- | --- | --- |
| *Eubacterium sp*. BAR | Barbaloin | Aloe-emodin 9-anthrone | 35% metabolite formation within 24 h, and reached maximum amount of aloe-emodin 9-anthrone after 36 h. | Che et al. 1991 |
| *Peptostreptococcus* YK-10 | Puerarin | Daidzein |  | Kim et al. 1998 |
| *Bacteroides sp*. MANG | Mangiferin | Norathyriol | Norathyriol reached maximum within 12 h of incubation time | Sanugul et al. 2005 |
| Strain PUE | Puerarin | Daidzein | 94.9% formation in 120 h | Jin et al. 2008 |
| *Lachnospiraceae* strain CG19-1 | Puerarin | Daidzein | Completely transformed 200 μM puerarin within 32 h | Braune and Blaut, 2011 |
|  | Homoorientin | Luteolin,  3-(3,4-hydroxyphenyl) propionic acid | 99% started substrate loss within 24 h |  |
|  | Vitexin | 3-(4-hydroxyphenyl) propionic acid | Completely transform within 24 h |  |
|  | Mangiferin | Norathyriol | 99% transform within 24h |  |
| *Enterococcus sp*. 45 | Orientin | Luteolin |  | Xu et al. 2014 |
| *Lactococcus sp.*MRG-IFC-1 | Puerarin | Daidzein | Transform completely in 40 min | Kim et al., 2015 |
|  | Vitexin | Apigenin | Not converted |  |
| *Enterococcus sp.* MRG-IFC-2 | Puerarin | Daidzein | Transform completely in 100 min |  |
|  | Vitexin | Apigenin | Not converted |  |
| *Enterococcus faecalis*  W12-1 | Puerarin, Homoorientin, Isoorientin | Daidzein,  Luteolin,  Apigenin | Not converted | Zheng et al. 2019 |
|  | Vitexin | Apigenin | Completely within 14h |  |
|  | Isovitexin | Apigenin | Completely in 6h |  |
|  | Orientin | Luteolin | Completely within 16h |  |

Braune A, Blaut M (2011) Deglycosylation of puerarin and other aromatic C-glucosides by a newly isolated human intestinal bacterium. *Environ Microbiol* 13(2):482-94.

Che QM, Akao T, Hattori M, Kobashi K, Namba T (1991) Isolation of a human intestinal bacterium capable of transforming barbaloin to aloe-emodin anthrone. *Planta Med* 57(1):15–19.

Jin JS, Nishihata T, Kakiuchi N, Hattori M (2008) Biotransformation of C-glucosyl isoflavone puerarin to estrogenic (3S)-equol in co-culture of two human intestinal bacteria. *Biol Pharm Bull* 31(8):1621–1625.

Kim DH, Jung EA, Sohng IS, Han JA, Kim TH, Han MJ (1998) Intestinal bacterial metabolism of flavonoids and its relation to some biological activities. *Arch Pharm Res* 21(1):17-23.

Kim M, Lee J, Han J (2015) Deglycosylation of isoflavone C-glycosides by newly isolated human intestinal bacteria. *J Sci Food Agric* 95(9): 1925–1931.

Sanugul K, Akao T, Li Y, Kakiuchi N, Nakamura N, Hattori M (2005) Isolation of a human intestinal bacterium that transforms mangiferin to norathyriol and inducibility of the enzyme that cleaves a C- glucosyl bond. *Biol Pharm Bull* 28(9):1672–1678.

Xu J, Qian D, Jiang S, Guo J, Shang EX, Duan JA, Yang J (2014) Application of ultra-performance liquid chromatography coupled with quadrupole time-of-flight mass spectrometry to determine the metabolites of orientin produced by human intestinal bacteria. *J Chromatogr B Anal Technol Biomed Life Sci* 944(3):123–127.

Zheng S, Geng D, Liu S, Wang Q, Liu S, Wang R (2019) A newly isolated human intestinal bacterium strain capable of deglycosylating flavone *C*-glycosides and its functional properties. *Microb Cell Fact* 18(1): 94.

Figure S1. Molecular structures of glycoside substrates used in the study.





Figure S2. ^1^H and ^13^C NMR spectra of methyl β-D-3-oxo-glucopyranoside in MeOH-*d*_4_.


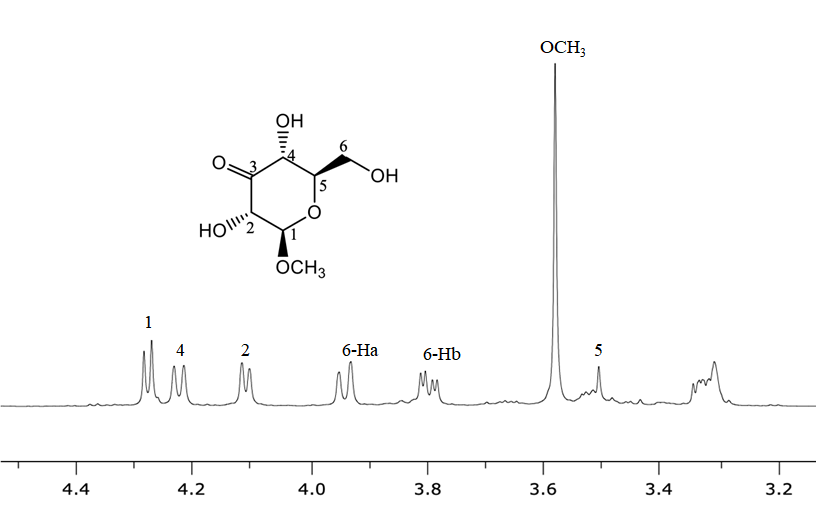


^1^H NMR (methanol-*d*_4_, 600MHz): δ 4.28 (1H, d, *J* = 7.8 Hz), 4.23 (1H; d; *J* = 10.2 Hz), 4.12 (1H, d, *J* = 7.8 Hz), 3.94 (dd, J=12, 6.0 Hz, C6-Ha), 3.80 (dd, J=12, 6.0 Hz, C6-Hb), 3.58 (3H, s, C1-OCH_3_), 3.51(1H, m).
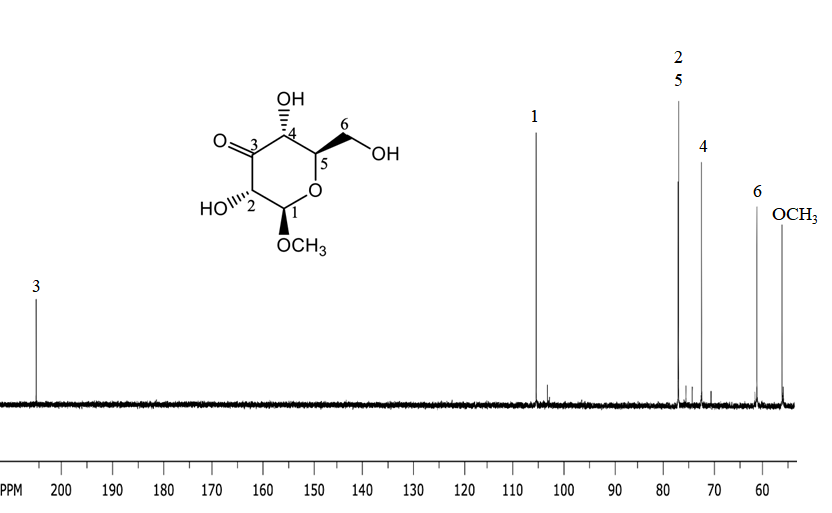


^13^C NMR (methanol-*d*_4_, 150 MHz): δ 205.6, 105.3, 76.9, 76.8, 72.2, 61.1, 56.1

Figure S3. ^1^H and ^13^C NMR spectra of 7-*O*-methylpuerarin in MeOH-*d*_4_ and DMF-*d*_7_, respectively.


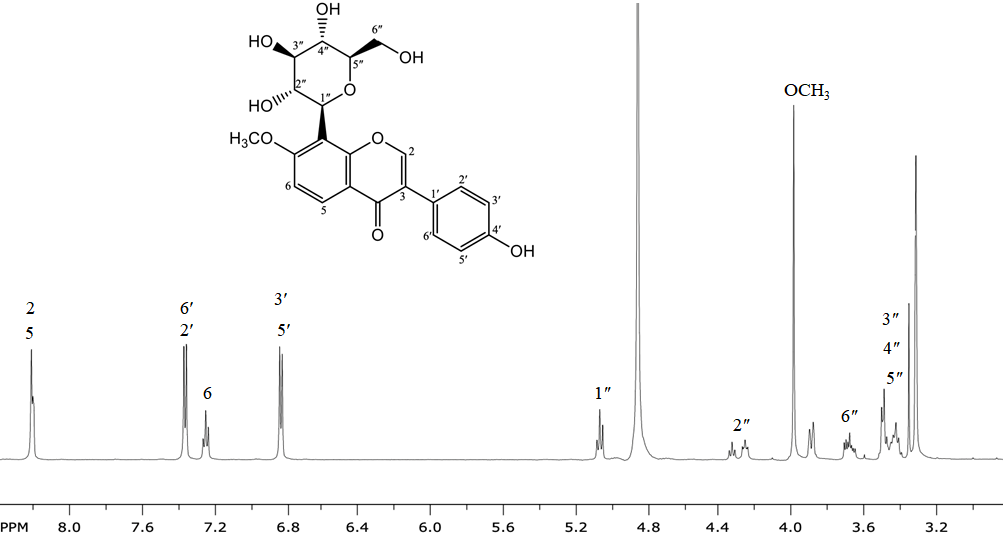


^1^H NMR of 7-*O*-methyl puerarin (methanol-*d*_4_, 600 MHz): δ 8.22 (2H, m), 7.37 (2H, d, *J* = 8.4 Hz), 7.27 (1H, m), 6.84 (2H, d, *J* = 8.4 Hz), 5.07 (1H, m), 4.3 (1H, m, t, *J* = 9 Hz), 3.98 (3H, s, OCH_3_), ), 3.62-3.90 (2H, m), 3.38-3.49 (3H, m)


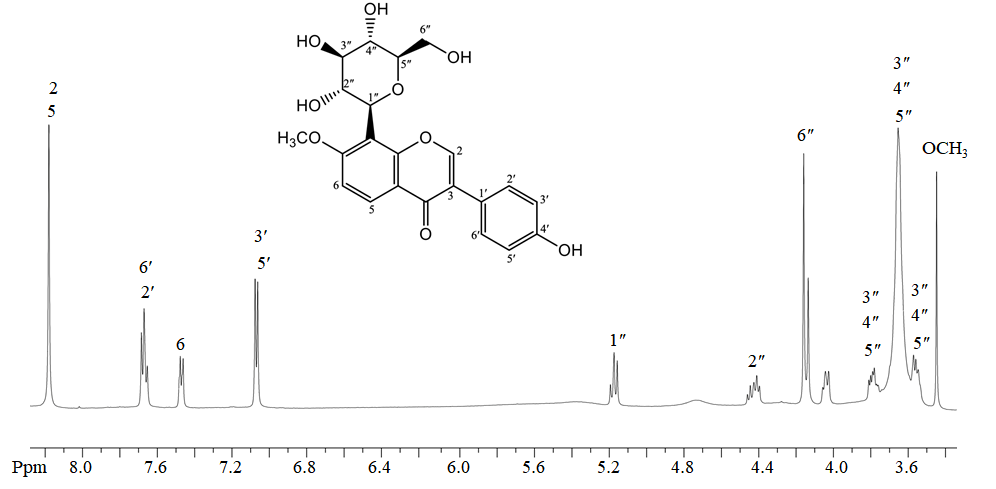


^1^H NMR (600 MHz, DMF-*d*_7_): 3.4 (*s*; 3H; OCH_3_), 3.7-3.8 (*br s*, 3H); 4.1 (*m*, 1H); 4.4 (*m*, 1H); 5.17 (*br s*, 1H); 7.0 (*d*, J=8.4 Hz; 3H); 7.5 (*m*, 2H); 7.7 (*m*, 1H, H-5); 8.19 (*s*, 1H).


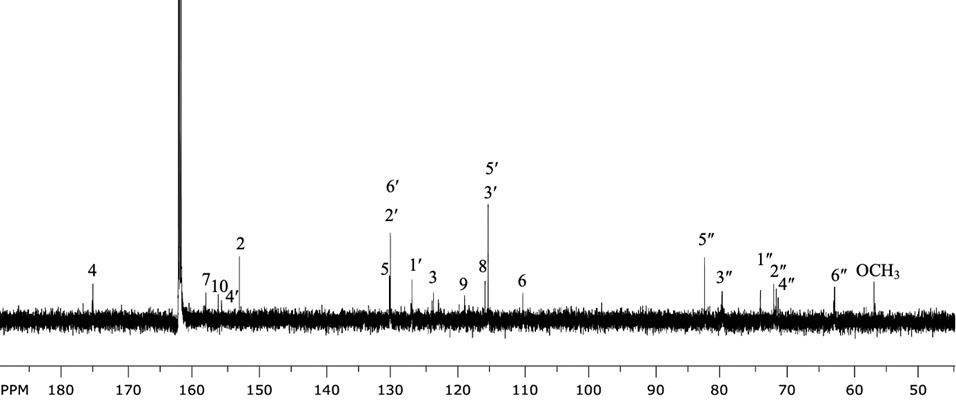


^13^C NMR spectra of 7-*O*-methylpuerarin (150 MHz; DMF-*d*_7_): δ 175.4, 158.3, 156.4, 155.9, 153.3, 130.4, 130.2, 127.0, 123.7, 119.0, 115.8, 115.3,110.1, 82.4, 79.7, 73.9, 71.9, 71.5, 62.6, 56.6.

Figure S4. DNA sequence of D3dgpA (OR238368.1). The four sequences in red represent different base from DgpA of PUE strain (BBG22493.1). But the amino acid sequence was identical.

atg agt aaa tta aaa att ggt att att ggt tgt ggt ggc atc gct aat cag aaa cat ttc

M S K L K I G I I G C G G I A N Q K H F 20

ccg gca tta aag aat aat gca gat ctg aat gaa atc gta gca ttt tgt gat att cag att

P A L K N N A D L N E I V A F C D I Q I 40

gac cgt gcc gag aaa gcg gca gca gaa ttt ggt gca gaa ggt gcg cag gta aca gct gac

D R A E K A A A E F G A E G A Q V T A D 60

tac aaa gag ctt ctg gct aac ccg gag gta gag gtt gta cat gtt tgt act cct aac gta

Y K E L L A N P E V E V V H V C T P N V 80

tcc cac agt gag att aca att gca gct ttt gaa gca ggc aag cac gtt tat tgt gaa aaa

S H S E I T I A A F E A G K H V Y C E K 100

cca atg tca cac agc aca gaa gaa gct gaa aag atg gtg gaa gca tgg aaa aag tcc ggc

P M S H S T E E A E K M V E A W K K S G 120

aag cag ttc acg att ggt tac cag aat cgt ttc cgt gaa gag gtt atg aat tta aag aag

K Q F T I G Y Q N R F R E E V M N L K K 140

tcc tgt gac aaa gga gaa ctt ggt gaa atc tac tat gga aaa gca cat gca gtt cgc cgc

S C D K G E L G E I Y Y G K A H A V R R 160

cgt gca gtt cct aca tgg ggc gta ttc atg gat aaa gaa gca cag ggc ggc gga cct ctt

R A V P T W G V F M D K E A Q G G G P L 180

atc gat atc gga aca cat gcc ctt gac att aca tta tgg tgt atg aac aac tac gat gta

I D I G T H A L D I T L W C M N N Y D V 200

gac agt gta act ggc tct gta ttc tac aag ctt gga cag aaa gaa aat gga cca gag ggt

D S V T G S V F Y K L G Q K E N G P E G 220

aat ctt ttc gga cca tgg gat cct aaa aca ttt gaa gta gaa gat tct gcg gtt gga ttt

N L F G P W D P K T F E V E D S A V G F 240

gtt aag atg aaa aat ggt gca aca atc ggt ctt gaa gca agt tgg gca att aac atg ctt

V K M K N G A T I G L E A S W A I N M L 260

gat tca aga gag gca tct aca aca ctt tgc gga aca gaa gcc ggt gca gag att cat tcc

D S R E A S T T L C G T E A G A E I H S 280

ggt atg agc tat cca aag aat gaa ctg att tat aat cgt gca cgc aat aat cag ctg atg

G M S Y P K N E L I Y N R A R N N Q L M 300

gaa gag act ctt tcc agc gtt gga agt att gct tac ttt gca ggt ggt gcc gga gaa gag

E E T L S S V G S I A Y F A G G A G E E 320

gga acc gtt gac aac cgt cag tgg ctt gaa gca att cag aat gga aca gaa cct ttg gtt

G T V D N R Q W L E A I Q N G T E P L V 340

aaa ccg gaa gaa gca ttg gca gta act aag att ttg gat gcg att tat aaa tcc gca aaa

K P E E A L A V T K I L D A I Y K S A K 460

aca aat gag aca att aaa ttc taa

T N E T I K F -

Figure S5. SDS-PAGE analysis of recombinant D3dgpA. Lane 1, marker proteins; lane 2, wash effluent; lane 3, D3dgpA effluent; lane 4, D3dgpA concentrate after ultrafiltration. Ni-NTA SDS-PAGE was carried out with 12% polyacrylamide gel and stained with Coomassie brilliant blue.


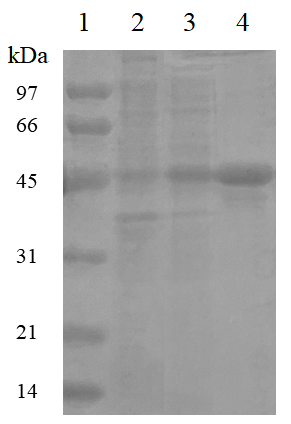


Fig S6. Methyl β-D-glucopyranoside was identified by LC-MS analysis of the reaction mixture.


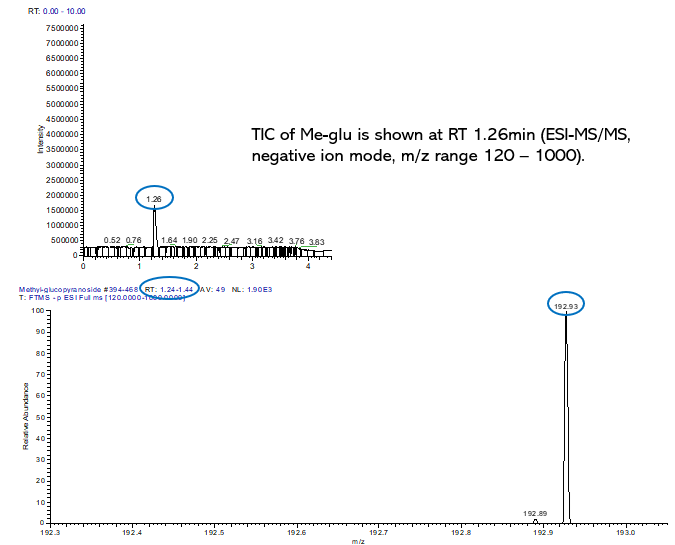


Figure S7. UV spectrum of purified D3dgpA (up) and HPLC analysis of NAD^+^ and NADH from the purified D3dgpA (down).


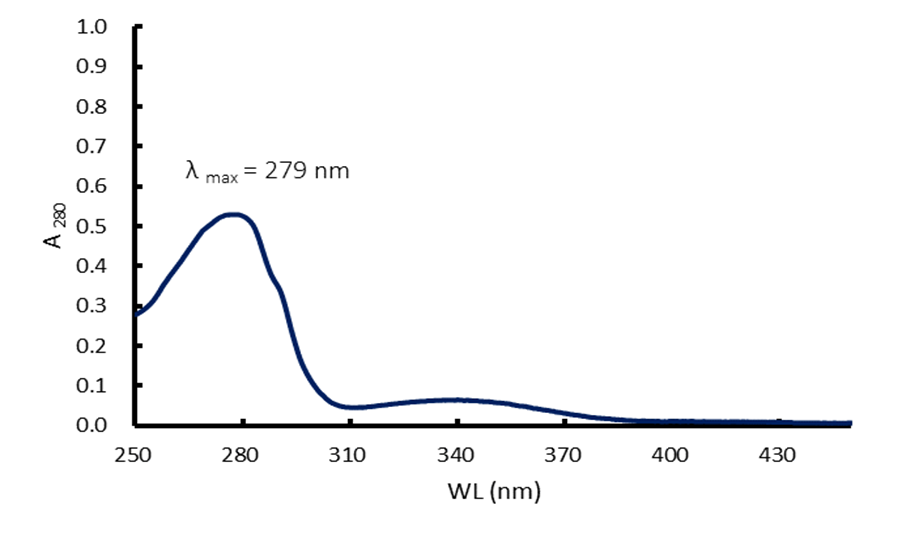

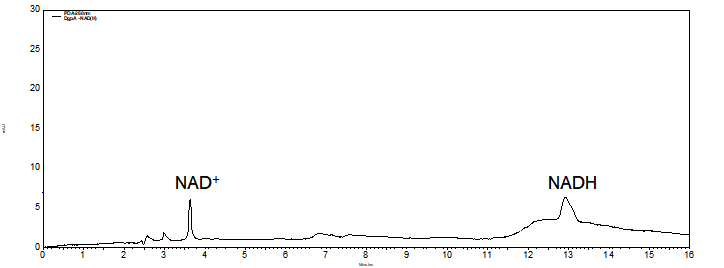


Figure S8. Relative activity of D3dgpA depending on [NAD^+^]. D3dgpA (2.5 μM) was added to the reaction mixture (100 μL) containing puerarin (1.0 mM) and methyl β-D-3-oxo-glucopyranoside (1.0 mM) in pH 8.0 (Tris 10.0 mM). The reaction was run for 30 min at 40°C with stirring (250 rpm).


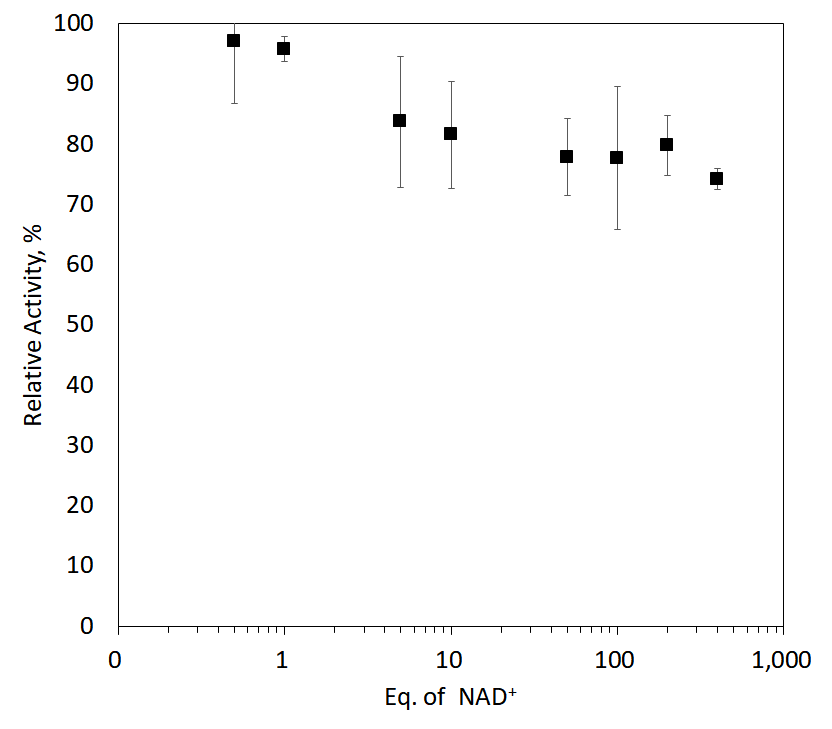


Figure S9. pH dependent activity of D3DgpA. For the pH-dependent activity of D3dgpA, puerarin oxidation was monitored at 37°C with a three-buffer system (10mM) (ref). D3dgpA was added to the solution containing 500 *eq* of puerarin and methyl β-D-3-oxo-glucopyranoside. Total volume of reaction mixture was 100μL and the reaction was run for 90min with stirring (250rpm).


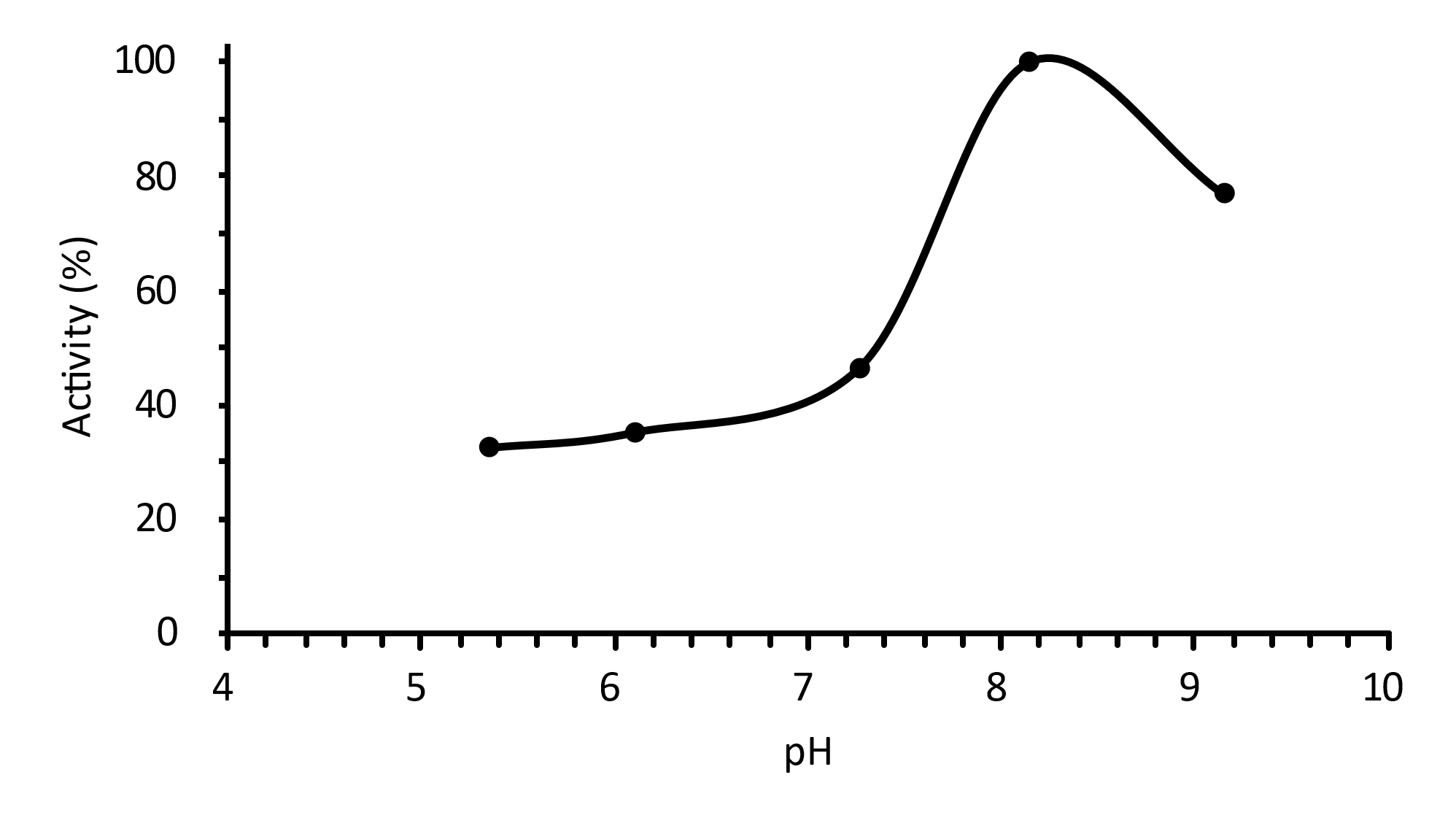


Figure S10. Stability of D3dgpA. The enzyme was stored at room temperature and the activity was measured in every 5 days. DgpA (5 μM) was reacted with 1.0 mM of puerarin in the presence of methyl β-D-3-oxo-glucopyranoside (1.0 mM), in pH 8.0 (10 mM Tris) for 30 minutes (250 rpm) at 37°C.


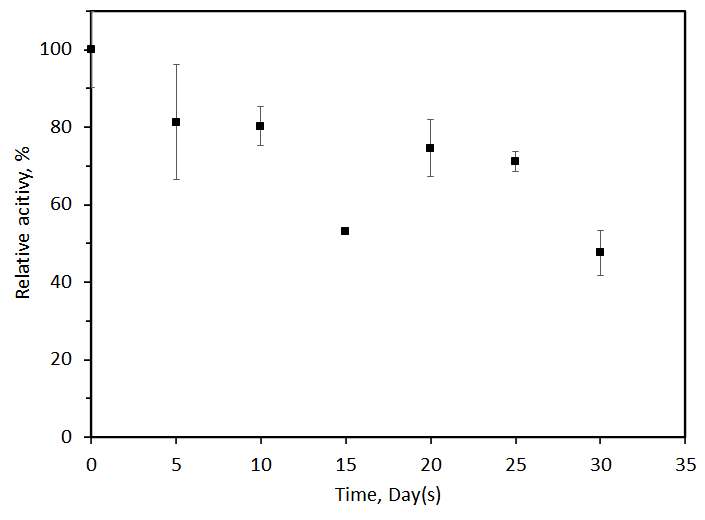


Figure S11. Michalis-Menten kinetics of D3dgpA.


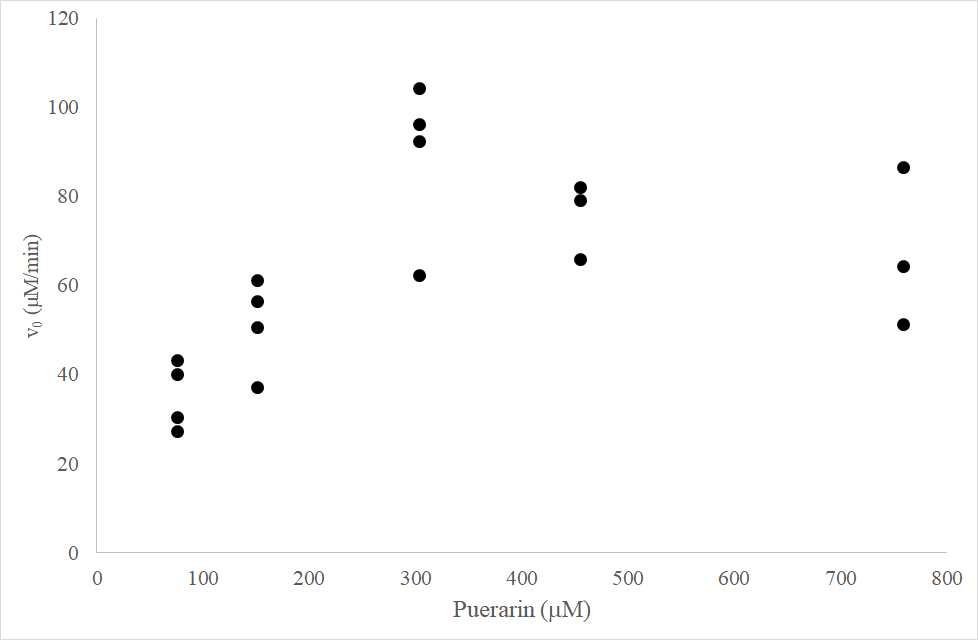


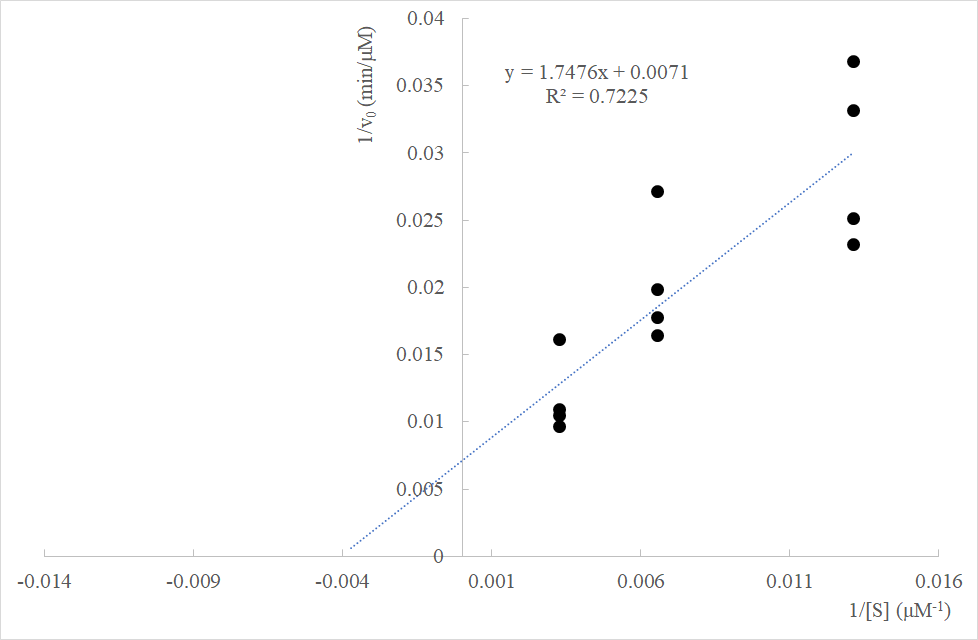


Figure S12. D3dgpA catalysis in the presence of the same amounts of puerarin and daidzin was monitored by HPLC analysis. D3dgpA (5μL, 137.5 nM final concentration) was added to 395 μL of pH 7.5 HEPES (5 mM) solution, containing puerarin and daidzin (137.5 nM each), methyl β-D-3-oxo-glucopyranoside (206 μM) at 36.5 °C, to initiate the reaction. The reaction mixture was stirred by 250 rpm, and 100μL of reaction mixture was taken in 3 min, 5 min, and 20 to be quenched by 5 % formic acid. The reaction product was filtered through the syringe filter before HPLC analysis (250 nm).

Figure S13. Conversion of *O*-glycosides by D3dgpA was monitored by HPLC analysis.


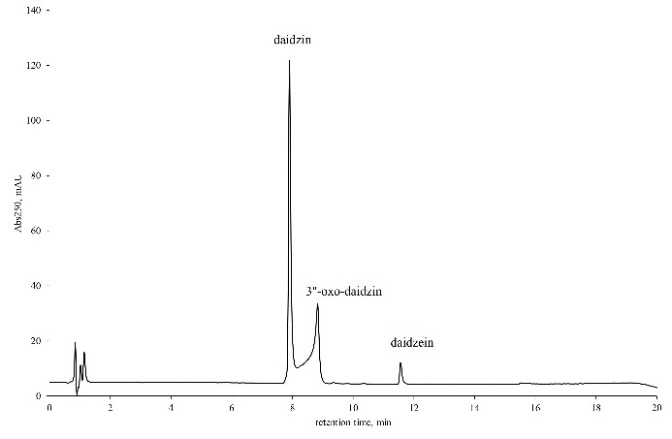

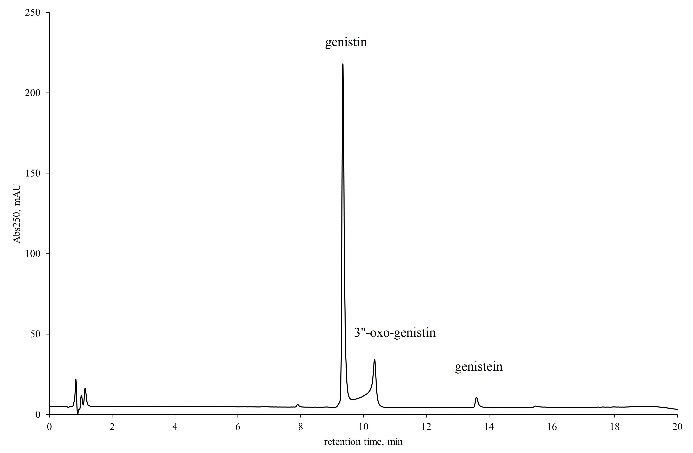

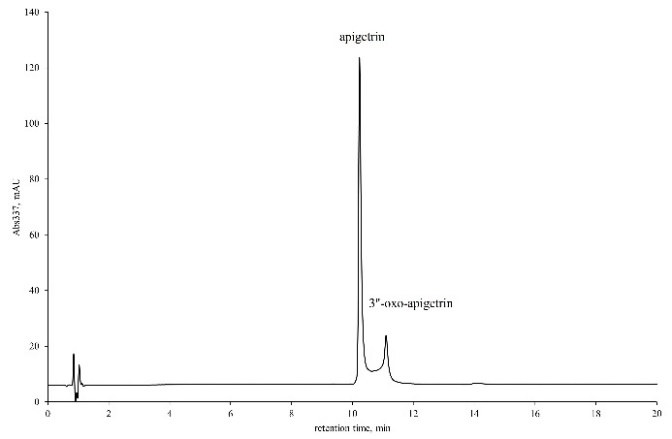

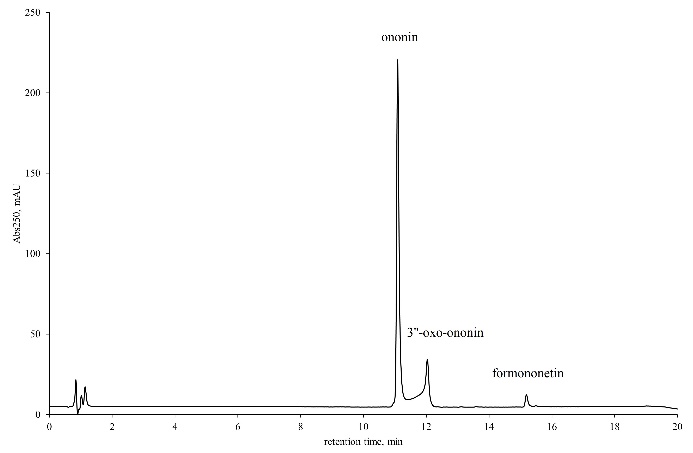

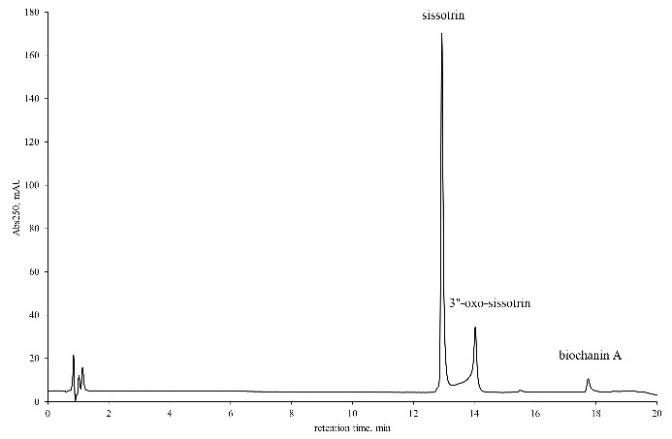

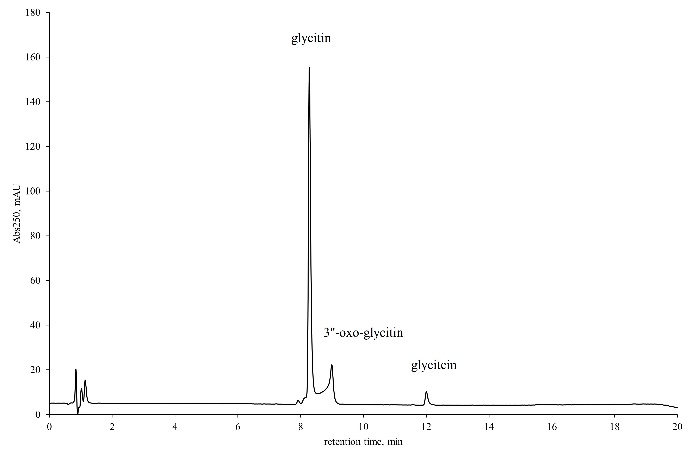

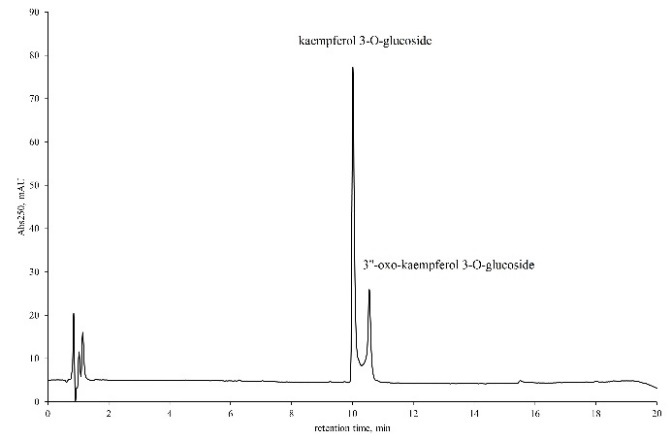

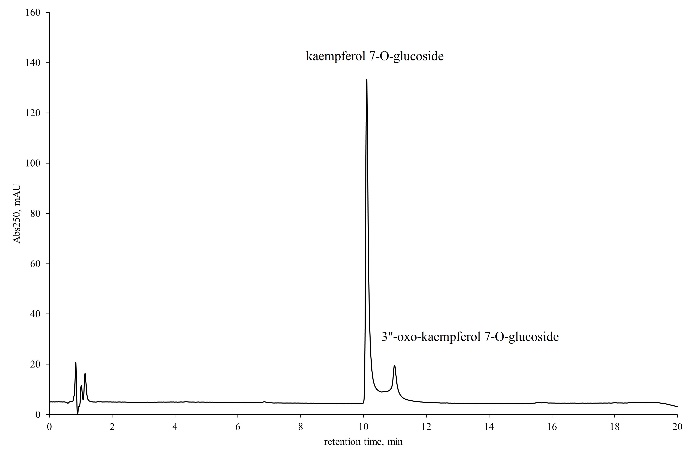

Supplement: Supplementary file 1 [file DataSheet1.docx]
